# Molecular cloning and functional characterization of the shikimate kinase gene from *Baphicacanthus cusia*

**DOI:** 10.3389/fpls.2025.1560891

**Published:** 2025-04-25

**Authors:** Yuxiang Huang, Hexin Tan, Qing Li, Xunxun Wu, Zhiying Guo, Junfeng Chen, Lei Zhang, Yong Diao

**Affiliations:** ^1^ School of Pharmacy, Quanzhou Medical College, Quanzhou, China; ^2^ School of Medicine, Huaqiao University, Quanzhou, China; ^3^ School of Pharmacy, Naval Medical University, Shanghai, China; ^4^ Institute of Chinese Materia Medica, Shanghai University of Traditional Chinese Medicine, Shanghai, China

**Keywords:** *Baphicacanthus cusia*, shikimate kinase, indole alkaloids, molecular cloning, functional characterization

## Abstract

*Baphicacanthus cusia* (Nee) Bremek, a perennial herbaceous plant with medicinal properties, has limited genomic insights regarding the genes involved in its indole alkaloid biosynthesis pathway. In this study, the *BcSK* gene was isolated and cloned from the transcriptome data of *B. cusia*. The full-length cDNA of *BcSK* is 1,657 bp, comprising a 265 bp 5’ UTR, a 507 bp 3’ UTR, and an 885 bp ORF encoding 295 amino acids. The exon-intron structure of *BcSK* consists of four exons and three introns. Bioinformatics and phylogenetic analyses revealed a high degree of homology between *BcSK* and its counterparts in various plant species. Quantitative real-time polymerase chain reaction (RT-qPCR) analysis showed that *BcSK* expression was significantly altered under abiotic stress conditions, including methyl jasmonate (MeJA), abscisic acid (ABA), and ultraviolet (UV) radiation. The gene was predominantly expressed in flowers compared to roots, stems, and leaves. Subcellular localization analysis indicated that *BcSK* is primarily expressed in chloroplasts, confirming that the conversion of shikimic acid to shikimate-3-phosphate occurs in this organelle. Prokaryotic expression and enzyme activity assays demonstrated that the heterologously expressed *BcSK* protein catalyzed the conversion of shikimic acid to shikimate-3-phosphate. Furthermore, the ectopic overexpression of *BcSK* in *Isatis indigotica* significantly enhanced the biosynthetic flux toward indole alkaloids, including indole, indigo, and indirubin. In conclusion, this study identifies and characterizes a novel *BcSK* gene, providing new insights and potential applications for the metabolic engineering of *B. cusia*.

## Introduction

*Baphicacanthus cusia* (Nee) Bremek, a prevalent member of the Acanthaceae family, primarily thrives in China. Its stems and leaves are commonly used for medicinal purposes (Ai, 2017). Renowned for its superior quality, the Fujian-originated product, known as “Jianqingdai” (*Indigo Naturalis*, IN), is considered one of Fujian’s authentic medicinal treasures (Xu, 2014). The root of *B. cusia*, recognized as Southern Banlangen (*Rhizoma et Radix Baphicacanthis Cusiae*, RRBC), is a prestigious traditional Chinese medicinal variety listed in the *Chinese Pharmacopoeia*, alongside Qingdai (National Pharmacopoeia Committee, 2020).

The primary active compounds in *B. cusia* belong to the indole alkaloid family, which includes indirubin, indigo, and isatin (Liu et al., 2009). Pharmacological studies have demonstrated that indigo exhibits potent immunomodulatory, antimicrobial, and hepatoprotective properties (Lee et al., 2019). Indirubin has shown promise as an antitumor agent or adjuvant therapy, exhibiting therapeutic efficacy against both transplanted animal tumors and human malignancies (Yu et al., 2021).

From a biological perspective, the genotype of medicinal plants plays a crucial role in determining their quality and efficacy (Huang et al., 2009). Despite being a key source of Qingdai, the genetic foundation of *B. cusia* remains poorly understood, and research on the synthesis pathways and metabolic networks of its indole alkaloids is limited. Genetic factors are essential for regulating metabolic pathways, and genetic engineering has emerged as a powerful approach for manipulating plant biosynthetic pathways (Huang et al., 2008, 2009). This technique enables the regulation of metabolic flux to enhance the production of pharmacologically active compounds. Functional genes are pivotal in plant genomics and play a fundamental role in secondary metabolic engineering (Farrokhi et al., 2006).

Indole alkaloids are synthesized from indole and tryptophan (O’Connor and Maresh, 2006). The incorporation of an indole nucleus, a widely recognized pharmacophore in indole alkaloids, results in a versatile heterocyclic structure with broad biological activity (Yu et al., 2021). This nucleus, derived from the shikimate pathway, serves as the structural backbone of indole alkaloids (Maeda and Dudareva, 2012). Notably, vinblastine and vincristine, two anticancer bisindole alkaloids in *Catharanthus roseus*, utilize tryptophan from the shikimate pathway as a key precursor (Sun et al., 2023). Genomic (Xu et al., 2020) and transcriptomic (Liu et al., 2023) studies on *B. cusia* have confirmed that the shikimate pathway is an integral component of its indole alkaloid biosynthesis.

Shikimate kinase (SK), a key enzyme in the fifth step of the shikimate pathway, irreversibly converts shikimate into shikimate-3-phosphate using ATP as a cofactor (Coracini and de Azevedo, 2014) (**Supplementary Figure S1**). This enzyme is also a target for drug design (Dadlani et al., 2022; Rios-Soto et al., 2021). SK has been extensively studied in microorganisms such as *Escherichia coli* (Millar et al., 1986) and *Mycobacterium tuberculosis* (Saidemberg et al., 2011; Rajput et al., 2023). In higher plants, SK genes have been cloned in rice (Kasai et al., 2005) and cabbage (Hu et al., 2021).

The biosynthesis and metabolism of indoles represent a crucial downstream branch of the shikimate pathway. Indigo and indirubin are dimeric indole compounds (**Supplementary Figure S1**). Thus, it can be inferred that the shikimate pathway plays a role in the biosynthesis of indole alkaloids in *B. cusia*, with *BcSK* potentially influencing this process.

In this study, the *BcSK* gene encoding shikimate kinase was identified from the *B. cusia* transcriptome. Its cloning, expression, and functional characterization were investigated to elucidate its biological role. These findings provide insights into the biosynthesis of pharmacologically active compounds in *B. cusia* and contribute to future research in secondary metabolic engineering and germplasm improvement.

## Materials and methods

### Sequence acquisition

The *BcSK* gene sequence was identified from the *B. cusia* transcriptome database (NCBI SRR4428209) using gene-specific primers SK-F and SK-R (**Supplementary Table S1**). The complete coding sequence of *BcSK* (**Supplementary Data Sheet S1**) was amplified via polymerase chain reaction (PCR) using KD Plus DNA Polymerase (TransGen Biotech, China). PCR amplification was conducted under stringent conditions: an initial denaturation at 98°C for 30 s, followed by 35 cycles of denaturation at 98°C for 10 s, annealing at 55°C for 30 s, and extension at 72°C for 1 min, with a final extension at 72°C for 7 min. The PCR products were then cloned into a pBlunt-Zero vector (TransGen Biotech, China) and transformed into *Trans1-T1* cells (TransGen Biotech, China) for further propagation and sequencing.

### Bioinformatic analysis

The cDNA sequences were analyzed using the Open Reading Frame (ORF) Finder to identify potential protein-coding regions. Vector NTI Advance (TM) 11.0 and ProtParam tools were employed to determine the isoelectric point, molecular weight, and solubility of the protein. Amino acid sequence alignment was performed using Clustal X2 software (version 1.83). Conserved motifs were identified using SMART.

To examine the phylogenetic relationships of *BcSK* across different plant species, a keyword search of the NCBI database was conducted. *BcSK* sequences from various species (Si XP_011100091.1, Eg XP_012845202.1, Nt NP_001312965.1, Si XP_011100896.1, Nt XP_009625951.1, Na XP_019257262.1, Nt XP_016441940.1, St XP_006362781.1, Ns XP_009762461.1, Sp XP_015071619.1, Sl NP_001234112.1, Ca XP_016552050.1, Dc XP_017229061.1, Vv XP_010652781.1, Vv NP_001268016.1, Pp XP_007205622.1, Pm XP_008232698.1, Gm XP_014634441.1, Cs XP_010416950.1, Cs XP_010429111.1, Cs XP_010472194.1, At NP_179785.2, At NP_001077937.1, Ca XP_004504021.1, Cs NP_001292691.1, Pt XP_002307130.2, At NP_195664.2, Va XP_017421154.1) were used to construct a phylogenetic tree using MEGA 5.0 with the neighbor-joining (NJ) algorithm and 1,000 bootstrap replications for robustness. All sequences are listed in **Supplementary Data Sheet S1**.

The SOPMA method was used to predict the secondary structure, while the tertiary structure of *BcSK* was modeled using Phyre 2. Domains, motifs, and active sites were identified using PredictProtein. The web addresses of relevant online analysis tools are listed in **Supplementary Table S2**.

### Plant materials and abiotic stress treatments

Specimens of *B. cusia* were collected from the Shufeng Farm, Fujian, China (25°25′N, 118°39′E). Tissue samples, including roots, stems, leaves, and flowers, were harvested from plants at the flowering stage. Six-month-old plants were subjected to stress treatments before flowering. Plants were transferred to flowerpots and exposed to UVB radiation (0.2 mW/cm²) for 3 h. Viable leaves were subsequently selected for analysis.

Additionally, the aerial parts were sprayed with 100 μM methyl jasmonate (MeJA) or 100 μM abscisic acid (ABA). Leaf samples were collected in triplicate at 0, 2, 4, 6, 8, 12, and 24 h post-treatment, resulting in 60 distinct samples: 12 organ-specific samples (roots, stems, leaves, flowers) and 48 stress-treated leaf samples (MeJA, ABA, UV radiation). After collection, samples were rapidly frozen in liquid nitrogen and stored at −80°C for further analysis.

### Total RNA extraction, cDNA synthesis, and RT-qPCR analysis

Frozen samples were homogenized in liquid nitrogen using a mortar and pestle. Total RNA was extracted using TRIzol reagent (Invitrogen, USA) and further purified with the Column Plant Total RNA Kit (TransGen Biotech, China) following the manufacturer’s protocol. RNA concentration was quantified at 260 nm using a NanoDrop 2000 spectrophotometer (Thermo, USA), and purity was assessed based on the 260/280 nm absorbance ratio. Only samples with an OD260/280 ratio between 1.9 and 2.2 and an OD260/230 ratio below 2.0 were selected for cDNA synthesis. RNA integrity was verified by agarose gel electrophoresis with ethidium bromide staining.

Genomic DNA was extracted from 100 mg of young leaves using the cetyltrimethyl ammonium bromide (CTAB) method and confirmed via agarose electrophoresis. First-strand cDNA synthesis was performed using the TransScript One-Step gDNA Removal and cDNA Synthesis SuperMix (TransGen Biotech, China), which included Oligo (dT) primer, gRemover, R-mix, and E-mix, with 1 μg of total RNA as the template. The reaction mixture (20 μL) was incubated at 42°C for 15 min, followed by enzyme inactivation at 85°C for 5 min. The resulting cDNA was stored at −20°C for future use.

Real-time PCR amplification was conducted in 96-well plates using a SYBR Green detection kit (TransGen Biotech, China) with a Thermal Cycler Dice TP800 (TaKaRa, Japan). Each 20-μL reaction contained 2.0 μL of template cDNA, 0.5 μL of each primer, 10.0 μL of 2× Top Green qPCR SuperMix, and 7.0 μL of ddH_2_O. A negative control was included, omitting the template. The amplification protocol consisted of an initial denaturation at 95°C for 30 s, followed by 40 cycles of denaturation at 95°C for 5 s and annealing at 60°C for 30 s. A melting curve analysis was performed over a temperature range of 60–95°C. Ct values were determined based on fluorescence thresholds.

To normalize target gene expression, *Bc18sRNA* (GenBank: GARR01001157.1) (Huang et al., 2017) was used as an internal control. Relative expression levels were calculated using the 2^−ΔΔCT method based on the average of three technical replicates. The primers used for RT-qPCR analysis are listed in **Supplementary Table S1**.

### Subcellular location of BcSK

Subcellular localization analysis using Plant-mPLoc predicted *BcSK* to be localized in the chloroplast. This prediction was further validated by a subcellular localization experiment involving *BcSK*-GFP fusion driven by the *CaMV* 35S promoter.

The experiment followed the methodology of Wang et al (Wang et al., 2018). The coding sequence of *BcSK* was amplified using gene-specific primers (**Supplementary Table S1**) and fused in-frame to the C-terminus of a green fluorescent protein (GFP) vector via the Gateway LR reaction (Invitrogen, USA). The recombinant vector (**Supplementary Figure S2**) was transformed into *Agrobacterium tumefaciens* strain GV3101, and bacterial cultures carrying the relevant plasmids were infiltrated into *Oryza sativa* protoplasts following the protocol of Liu et al (Liu et al., 2007). Fluorescent signals from the *BcSK*-GFP fusion protein were detected using a confocal laser scanning microscope (LSM 800, Zeiss).

### Protein prokaryotic expression and purification

The *BcSK* gene was first transformed into *Trans1-T1* cells for amplification. The PCR-derived products were digested with *BamHI* and *XhoI* (TaKaRa, Japan) and ligated into the *pGEX* expression vector (Novagen, Madison, WI, USA). The recombinant plasmid (**Supplementary Figure S3**) was then transformed into *E. coli* BL21 (DE3) and cultured in Luria–Bertani (LB) medium containing ampicillin (100 μg/mL) at 37°C until the OD600 reached 0.6. Protein expression was induced by adding 1.0 mM isopropyl β-D-1-thiogalactopyranoside (IPTG), and the culture was incubated at 16°C, 80 rpm, for 24 h.

Cells were harvested by centrifugation at 4°C, 5,000 rpm, for 10 min and resuspended in a lysis buffer containing 50 mM NaH_2_PO_4_ (pH 8.0), 10 mM Tris-HCl (pH 8.0), and 100 mM NaCl. Sonication was performed to reduce viscosity. The recombinant protein was purified using Bio-Scale Mini Profinity GST Cartridges. The components were separated by 10% SDS-PAGE, distinguishing the supernatant, pellet, and purified product. Proteins were visualized using Coomassie Brilliant Blue (CBB) staining, and images were captured with an Amersham Imager 600 (AI600). Western blotting was performed to confirm the expression of GST-fused *BcSK* using an anti-GST rabbit monoclonal antibody (Cell Signaling Technology), followed by a secondary anti-rabbit IgG HRP-linked antibody.

### Protein quantification and enzyme activity detection

Protein concentration was determined using the Bradford Protein Concentration Kit. The enzymatic activity of BcSK was assessed by coupling ADP release to sequential reactions catalyzed by pyruvate kinase (PK) and lactate dehydrogenase (LDH) (Millar et al., 1986; Rosado et al., 2013). The activity of shikimate-mediated reactions was quantified by monitoring the decrease in absorbance at 340 nm at 15 s intervals due to NADH oxidation.

Enzymatic assays were conducted at 298 K in 96-well polystyrene plates (Costar) using a BioTek Synergy 4 plate reader. The reaction mixture (200 μL) contained 100 mM Tris-HCl (pH 7.5), 5 mM MgCl_2_, 50 mM KCl, 1.6 mM ATP, 0.2 mM NADH, 1.5 mM phosphoenolpyruvate, 6 U/mL PK, and 5 U/mL LDH. The reaction was initiated by adding 50 nM purified BcSK, and kinetic parameters were determined by measuring initial reaction rates across a range of shikimate concentrations (10–1,200 mM). Final kinetic parameters were calculated using non-linear regression analysis in GraphPad Prism (GraphPad Software Inc.). All assays were performed in triplicate.

The activity of BcSK was calculated using the following formula:


$$\begin{array}{c} \text{Enzyme activity }\left(\text{U}/\text{gprot}\right)\;\\ =△\text{A}/\text{min}/0.001/\text{protein concentration} \end{array}$$

### Construction of *BcSK* overexpression vector and *I. indigotica* transformation

The full-length coding sequence of *BcSK-PHB* was amplified from leaf-derived cDNA using primers PHB-SK-F and PHB-SK-R (**Supplementary Table S1**). The amplified fragment was cloned into the modified binary vector PHB-Flag at the *BamHI* and *SpeI* restriction sites. This vector contained two *CaMV* 35S promoters, serving as an efficient overexpression tool (Masani et al., 2009).

**Figure 1:**
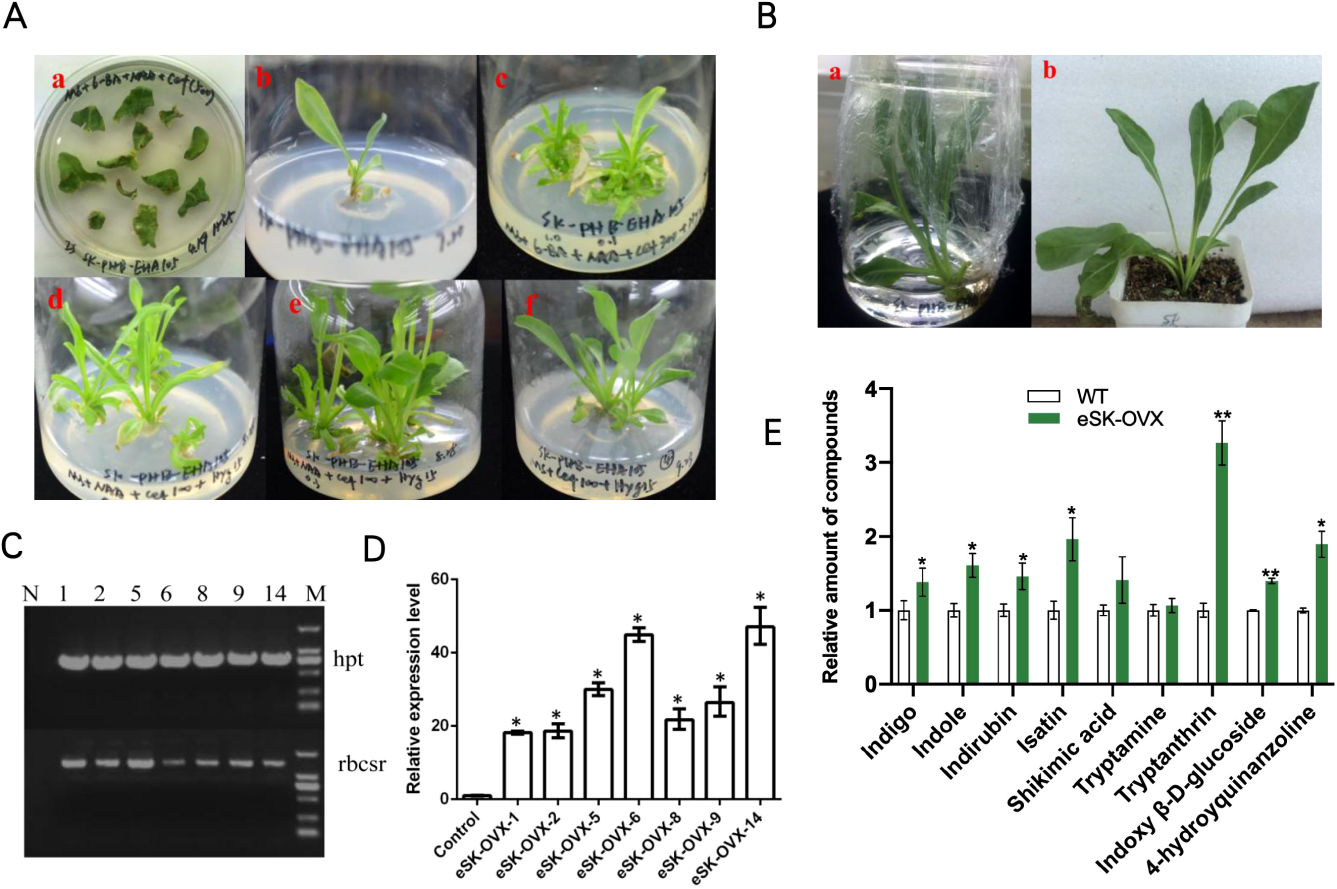
Regenerated Plants of OVX-*BcSK* in *I indigotica.* **(A)** Induction and Culture of Regenerated *I indigotica* Plants at Different Stages. (a) *Agrobacterium tumefaciens* EHA105 strains carrying plasmids were used to infect sterilized leaf explants, leading to callus formation. (b) Newly formed shoots were transferred to MS medium supplemented with plant auxins. (c–f) Positive regenerated plants containing the *EHA105-OVX-BcSK* vector exhibited robust growth on MS solid medium supplemented with cefotaxime and hygromycin. **(B)** Transferring Positive Regenerated Plants to Water and Soil for Cultivation. Regenerated plants harboring the *EHA105-OVX-BcSK* vector were successfully transferred to water or soil for further cultivation. **(C)** Molecular Identification of Transgenic Plants Lane 1: Engineered strain (positive control). Lane 2–8: *OVX-BcSK* transgenic plants. Lane 9: DNA size marker. The *hpt* primer was used to verify hygromycin resistance. **(D)** Expression Analysis of *BcSK* RT-qPCR analysis of *BcSK* transcript abundance in *OVX-BcSK* transgenic plants. Control: *B. cusia* leaf. **(E)** Metabolite Analysis of Transgenic Plants Metabolite quantification was conducted to evaluate indole alkaloid levels. Data are presented as mean ± SEM; *P< 0.05, **P< 0.01 compared to the control.

*Agrobacterium tumefaciens* strain C58C1 harboring *BcSK-PHB* (**Supplementary Figure S4**) and the PHB vector were used to infect *I. indigotica* leaf explants (Xiao et al., 2015). After two days of dark incubation without antibiotics, the explants were transferred to half-strength Murashige and Skoog (1/2 MS) solid medium supplemented with a stepwise reduction of cefotaxime (250, 100, 0 mg·L^-1^) and 10 mg·L^-1^ hygromycin (**Figure 1A**). After 45 days, the hairy roots were harvested for DNA and RNA extraction and subsequent metabolite analysis. Transgenic *BcSK-PHB* lines were designated as “cSK-OVX,” while non-transformed C58C1 served as the wild-type (WT) control.

Regenerated plants were obtained using *A. tumefaciens* strain EHA105 following a similar infection protocol as for hairy root cultures. However, the culture medium was supplemented with 6-benzylaminopurine (6-BA, 1.0 mg·L^-1^) and α-naphthylacetic acid (NAA, 0.1 mg·L^-1^), and cefotaxime concentrations were gradually reduced (500, 300, 100, 0 mg·L^-1^). Once budding and rooting occurred, growth hormones were gradually removed, and the plants were transferred from water culture to soil. When tissue culture seedlings reached 10 cm in height, leaves were harvested and frozen in liquid nitrogen for further analysis. Transgenic *BcSK-PHB* lines were labeled “eSK-OVX,” whereas non-transformed EHA105 served as the WT control.

Each experiment was conducted in triplicate to ensure biological reproducibility.

### Analysis of transgenic products

Polymerase chain reaction (PCR) was used to identify transgenic regenerated plants and hairy roots (Yadav et al., 1982). Genomic DNA was extracted from confirmed positive transgenic samples using the cetyltrimethyl ammonium bromide (CTAB) method. Regenerated plants were screened for the *hpt* resistance gene fragment of the PHB-Flag vector, while the *rbcsr* sequence from the *pC1300-pHANNIBAL* vector was also utilized for identification. Additionally, the presence of the target gene fragment was verified.

For hairy roots, in addition to confirming the *hpt* resistance and target gene fragments, *rolB* and *rolC* fragments from the Ri plasmid of *Agrobacterium rhizogenes* C58C1 were also identified.

Quantitative real-time PCR (RT-qPCR) was performed to measure *BcSK* expression levels in all verified positive lines, using *IiActin* as an internal reference. Primer sequences used for detection are provided in **Supplementary Table S1**. At least three independent control lines were analyzed, and their average value was used as the control benchmark.

### Extraction and determination of metabolite concentrations

Harvested transgenic hairy roots were dried at 40°C for 48 h and ground into a fine powder, while plant leaves were pulverized under liquid nitrogen. A 100 mg sample of powder was extracted via ultrasonication for 1 h using 5 mL of methanol and trichloromethane (1:1). The supernatant was transferred, and the precipitate was re-extracted. The combined extracts were filtered through a 0.22 μm microporous membrane. A 5 mL aliquot of the supernatant was dried and redissolved in 200 μL of methanol.

Metabolite quantification was performed using high-performance liquid chromatography-mass spectrometry (HPLC-MS) on the Agilent 1260 Infinity platform (Agilent, USA). HPLC analysis utilized a Poroshell 120 EC-C18 column (3.0 × 150 mm, 2.7 μm) with a flow rate of 0.4 mL/min at 25°C. The injection volume was 3 μL, and elution was performed in gradient mode (**Supplementary Table S2**). The mobile phase consisted of 5 mM ammonium acetate (Phase A) and 5 mM ammonium acetate in acetonitrile/methanol (Phase B, HPLC grade).

Mass spectrometry employed an electrospray ionization (ESI) source with a nebulizer gas pressure of 45 psi, drying gas temperature of 350°C, and drying gas flow rate of 10 L/min. The capillary voltage was set to 4000 V (+) and 3000 V (−). Data acquisition and analysis were conducted using the Mass Hunter software control system and processing workstation (Agilent, USA). Multiple reaction monitoring (MRM) mode was used for target compound detection. All required standards were obtained from Sigma-Aldrich (St. Louis, MO, USA).

### Statistical analysis

All experiments were conducted in triplicate, and data were analyzed using GraphPad Prism 8.0 software. Results are presented as mean ± SEM.

For multiple comparisons versus the control, one-way ANOVA followed by Scheffé’s *post hoc* test was performed. For comparisons between two groups, a Student’s *t*-test was used. Statistical significance was set at *p*< 0.05 and *p*< 0.01.

## Results

### Isolation, gene structure, and bioinformatics analysis

**Figure 2:**
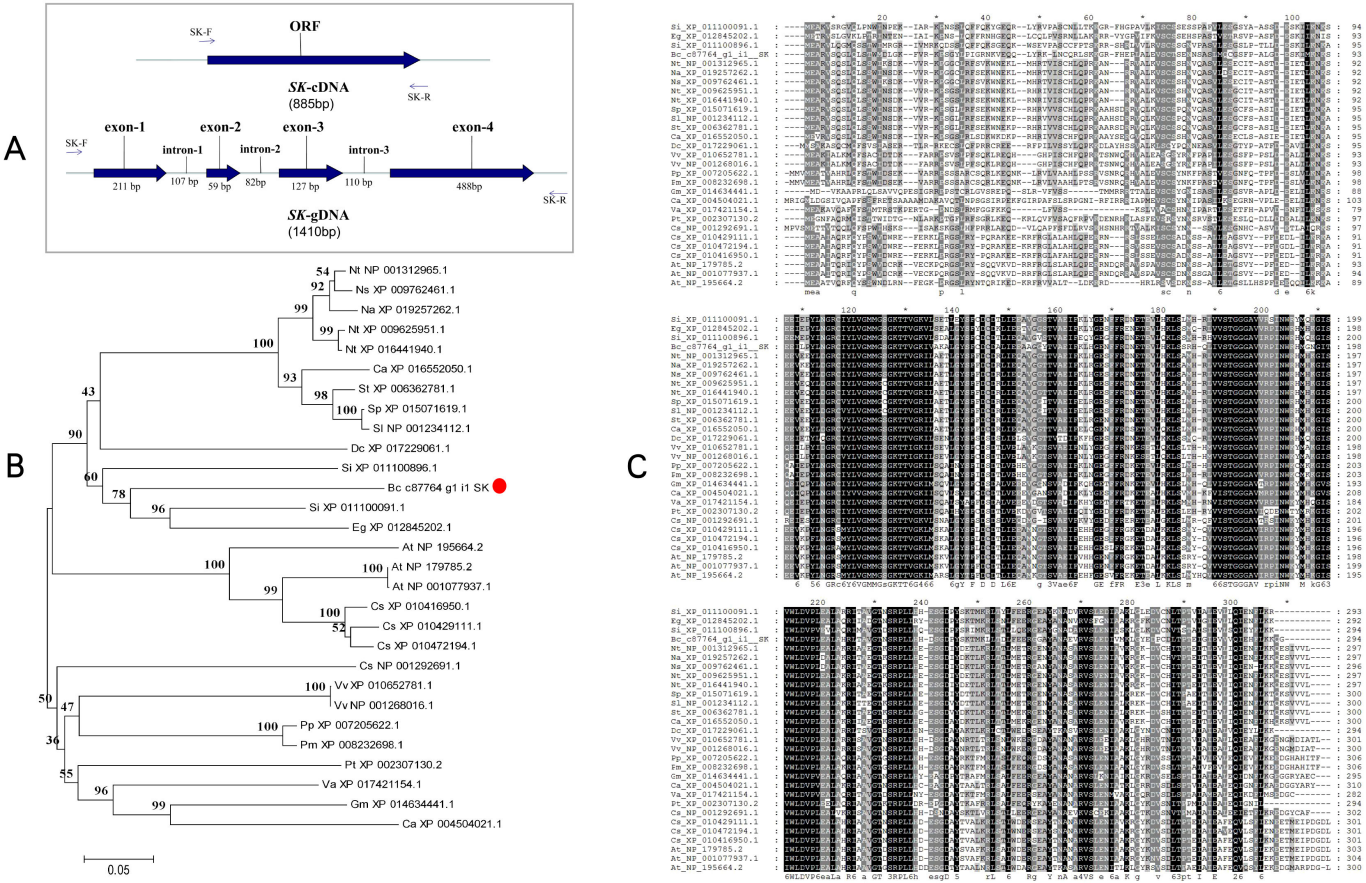
Schematic diagram of the *BcSK* gene and its bioinformatics analysis. **(A)** cDNA and gDNA structure of *BcSK*. **(B)** Phylogenetic tree of the *BcSK* protein family from 28 plant species, constructed using MEGA 5.0 with the neighbor-joining method. **(C)** Homology comparison of *BcSK* amino acid sequences across 28 plant species.

The *BcSK* cDNA consists of an 885 bp open reading frame (ORF) encoding a 295-amino acid peptide with a predicted molecular weight of 32.70 kDa. The isoelectric point (pI) is 6.83, indicating that *BcSK* is slightly acidic. Analysis of its exon-intron structure revealed four exons (211 bp, 59 bp, 127 bp, and 488 bp from the 5′ to 3′ end) and three introns (107 bp, 82 bp, and 110 bp, respectively) (**Figure 2A**).

The *BcSK* protein contains a distinct hydrophobic region with a value of −0.295, adjacent to a hydrophilic domain (**Supplementary Figure S5C**). Structural predictions indicate that *BcSK* adopts a stable conformation without a signal peptide, though it may contain a minor transmembrane topology segment (**Supplementary Figure S5B**). The secondary structure is predicted to consist of 45.58% alpha helices, 7.48% beta-turns, 34.35% random coils, and 12.59% extended strands (**Supplementary Figure S5A**). Moreover, *BcSK* possesses a conserved active structural domain characteristic of shikimate kinase (SK) enzymes, spanning amino acids 111–270 (**Supplementary Figure S6A, B**).

A phylogenetic tree was constructed based on a comparative analysis of *B. cusia* and 28 other plant species, revealing that *BcSK* shares a clade with *Sesamum indicum* (**Figure 2B**). Amino acid sequence homology analysis demonstrated high similarity between *BcSK* and SK proteins from *S. indicum, Erythranthe guttata, Nicotiana tabacum*, and *Arabidopsis thaliana* (**Figure 2C**).

### Induction and expression patterns

**Figure 3:**
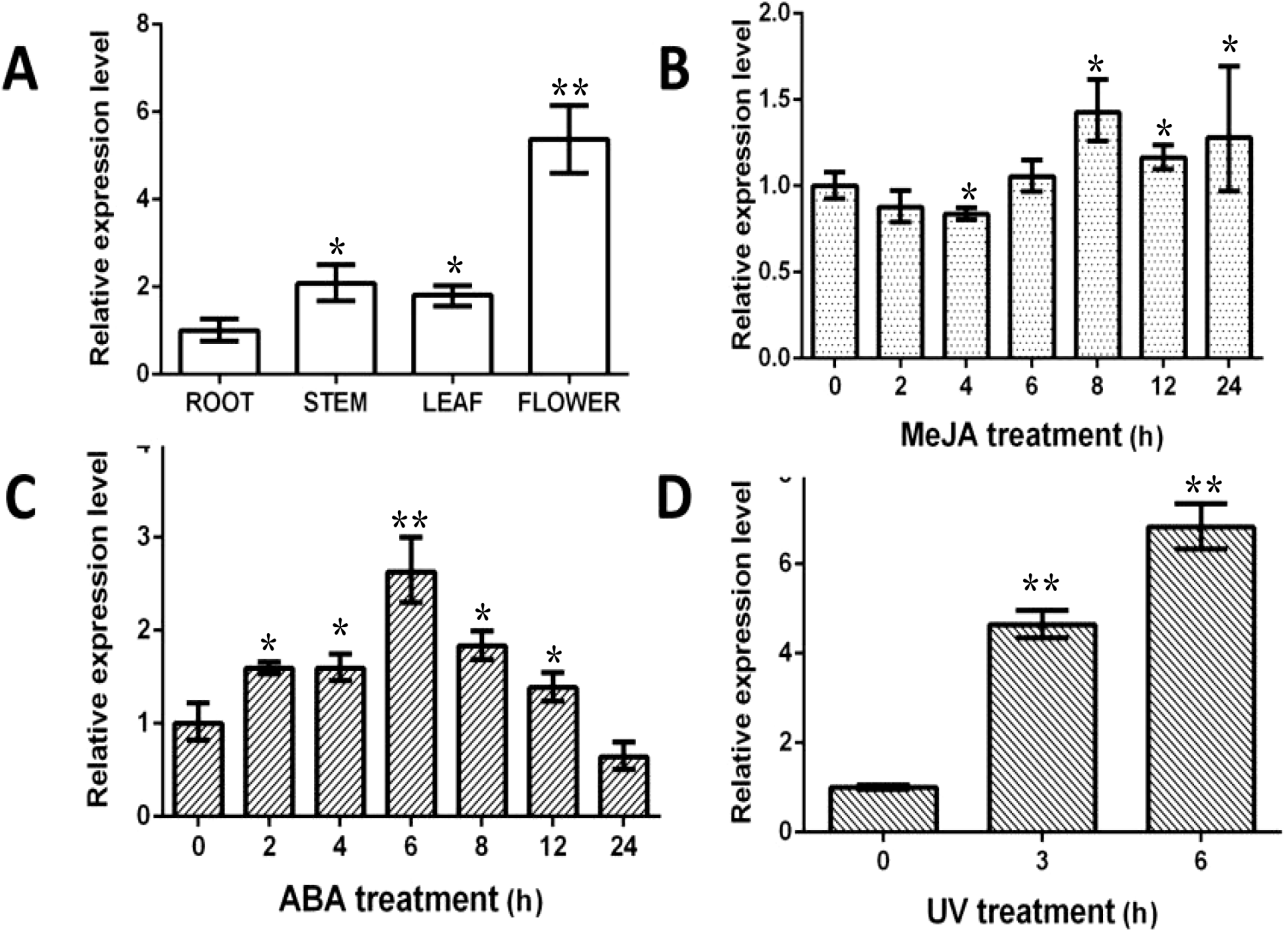
Expression profiles of *BcSK* in different *B cusia* organs: **(A)** Basal expression levels in various tissues. **(B)** Induction by MeJA. **(C)** Induction by ABA. **(D)** Response to UV stress. Data are presented as mean ± SEM; *P< 0.05, **P< 0.01 compared to the control.

To investigate *BcSK* expression patterns, RNA was extracted from different *B. cusia* tissues, including roots, stems, leaves, and flowers, for RT-qPCR analysis. The results indicated that *BcSK* was expressed in all tissues, with the highest expression in flowers, followed by stems and leaves, and the lowest in roots (**Figure 3A**).

RT-qPCR analysis further demonstrated that *BcSK* expression was significantly upregulated in response to methyl jasmonate (MeJA), abscisic acid (ABA), and ultraviolet (UV) stress, exhibiting phytohormone-specific and time-dependent regulatory patterns (**Figures 3B–D**). Upon MeJA treatment, *BcSK* expression peaked at 8 hours, briefly declined, and then gradually increased until 24 hours. ABA exposure led to a marked 2.63-fold upregulation at 6 hours, followed by a gradual decline while remaining above baseline at 24 hours. Under UV stress, *BcSK* expression surged rapidly, reaching a substantial 6.82-fold increase at 6 hours compared to the control, suggesting a strong induction response.

### Subcellular localization characteristics

**Figure 4:**
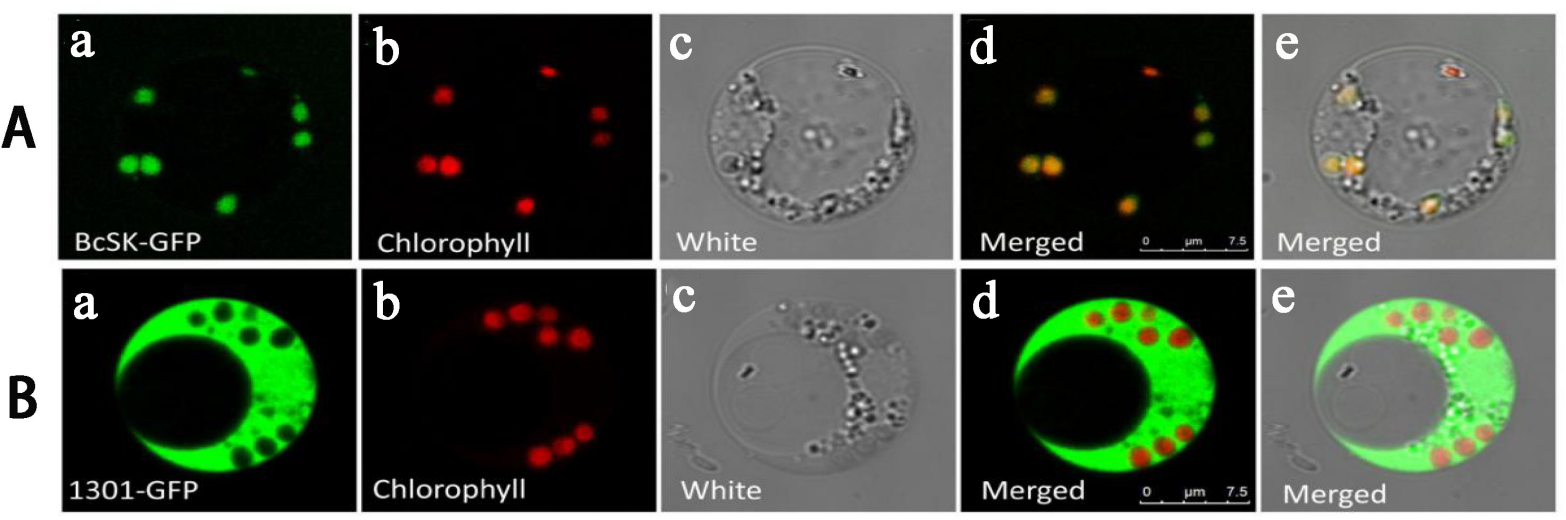
Subcellular localization of *BcSK*: **(A)** *BcSK*-GFP transiently expressed in rice protoplasts. **(B)** Control contrast images. (A) GFP fluorescence. (B) Chlorophyll autofluorescence. (C) Bright-field image. (D) Merged image of (A) and (B). (E) Merged image under bright-field conditions. Scale bar = 5 nm.

To verify the subcellular localization of *BcSK*, the gene was fused with a green fluorescent protein (GFP) tag and expressed under the *CaMV* 35S promoter. The construct was transiently introduced into *Oryza sativa* protoplasts, with an empty *pCAMBIA1301*-GFP vector serving as a control (**Figure 4**). In protoplasts expressing the GFP control, fluorescence was distributed throughout the entire cell (**Figure 4II**A). In contrast, fluorescence from *BcSK*-GFP was exclusively detected within the chloroplast (**Figure 4I**A–E), indicating chloroplast localization. Additionally, overlapping signals from GFP (**Figure 4I**A) and RFP (**Figure 4I**B) (**Figure 4I**D) suggested an association of *BcSK*-GFP with the endoplasmic reticulum (ER). These findings confirm that *BcSK* is primarily localized in the chloroplast, consistent with prior predictions (**Figure 4I**A).

### Prokaryotic expression and enzymatic activity

**Figure 5:**
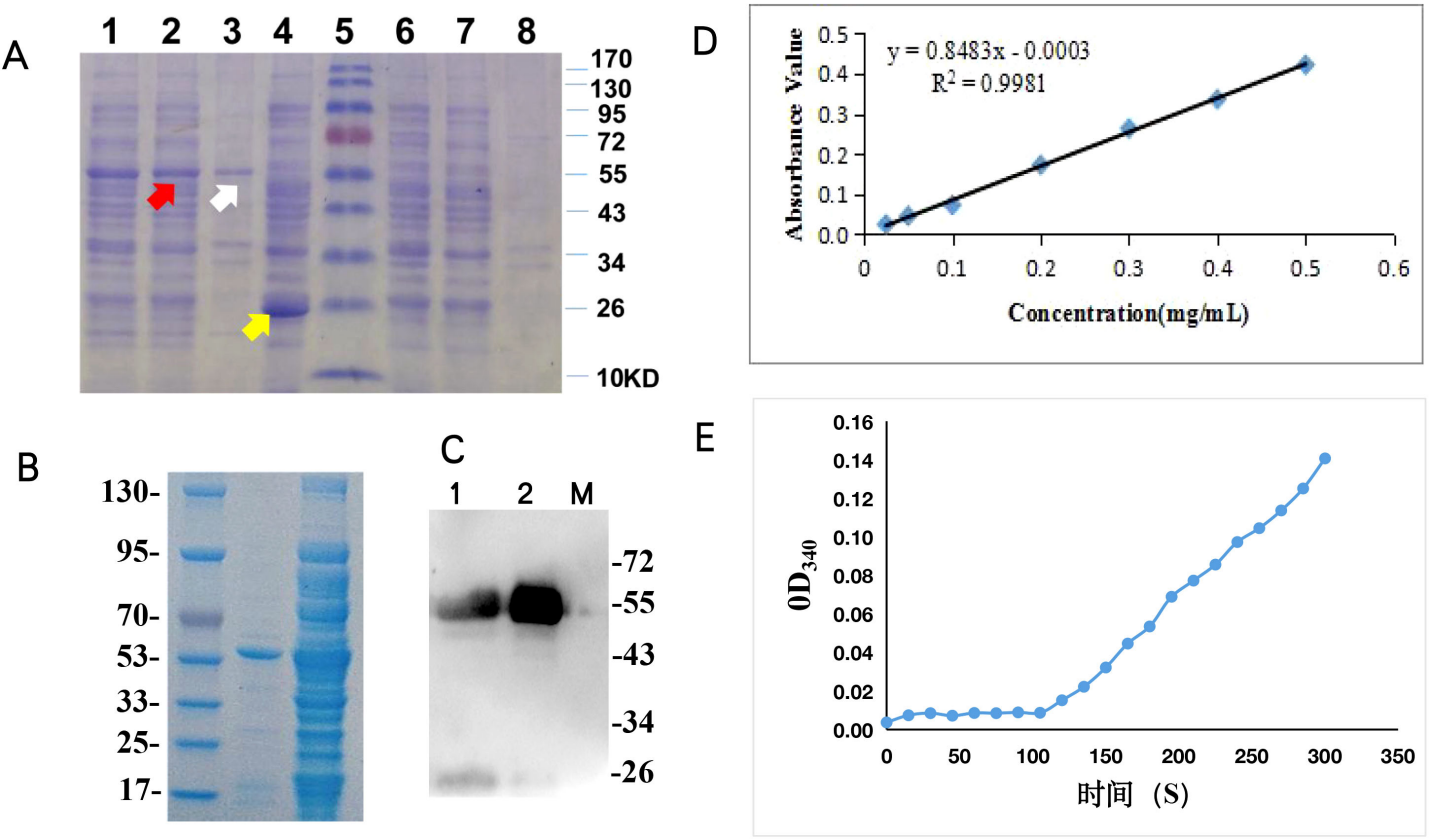
Functional characterization of *BcSK in vitro*: **(A)** SDS-PAGE analysis of *BcSK*-pGEX fusion protein. Lane 1: Crude enzyme extract from *BcSK*-pGEX cell lysate. Lane 2: Supernatant of *BcSK*-pGEX cell lysate. Lane 3: Precipitate of *BcSK*-pGEX cell lysate. Lane 4: Crude enzyme extract from *pGEX-4T-1* cell lysate after IPTG induction. Lane 5: Protein molecular weight marker. Lane 6: Crude enzyme extract from *pGEX-4T-1* cell lysate without IPTG induction. Lane 7: Supernatant of *pGEX-4T-1* cell lysate without IPTG induction. Lane 8: Precipitate of *pGEX-4T-1* cell lysate without IPTG induction. **(B)** SDS-PAGE analysis of purified *BcSK*-pGEX fusion protein. Lane 1: Protein molecular weight marker. Lane 2: Purified *BcSK*-pGEX protein. Lane 3: Supernatant of *BcSK*-pGEX cell lysate. **(C)** Western blot analysis of *BcSK*-pGEX. Lane 1: Supernatant of *BcSK*-pGEX cell lysate. Lane 2: Purified *BcSK*-pGEX protein. Lane 3: Protein molecular weight marker. **(D)** Calibration curve of protein concentration. **(E)** Enzyme kinetic characterization of *BcSK in vitro*.

To assess *BcSK* function, the gene was cloned into the *pGEX-4T-1* prokaryotic expression vector with a GST-tag for detection. The construct was introduced into *E. coli* BL21 (DE3) and confirmed via PCR (**Supplementary Figure S3**) and DNA sequencing. The recombinant *BcSK* protein was purified and analyzed by SDS-PAGE (**Figure 5**).

The expected molecular mass of the *BcSK*-GST fusion protein was approximately 55.7 kDa, comprising BcSK (32.7 kDa) and the GST-tag (23 kDa) (**Figure 5A**). Western blot analysis confirmed immunoreactivity of the purified *BcSK*-GST protein against anti-2GST antibodies (**Figure 5B**). The Bradford assay determined the protein concentration, generating a standard curve with the equation *Y = 0.8483x − 0.0003* (*R² = 0.9981*, **Figure 5C**). The final concentration of purified *BcSK*-GST protein was 8.4 mg/mL.

BcSK catalyzes the phosphorylation of shikimic acid to form shikimate-3-phosphate. Enzyme activity was assessed using a coupled reaction system, measuring OD340 of NADH every 15 s. A linear correlation was observed (**Figure 5D**) after 105 s, and OD340 values recorded within 1 min yielded an average change of ΔOD340 = 0.0358. This corresponded to a BcSK enzyme activity of 4.26 U/g. Changes in OD340 confirmed the redox reaction, where NADH was converted to NADPH, indirectly validating enzymatic activity following the addition of *BcSK*-GST. The product was subsequently used as a substrate in a secondary reaction catalyzed by lactate dehydrogenase, further confirming that the *BcSK*-GST fusion protein was functionally active and capable of catalyzing shikimic acid phosphorylation.

### Overexpression of *BcSK* increased the content of indole alkaloids in *I. indigotica*

Transgenic plants overexpressing *BcSK* (*BcSK*-OVX) were successfully generated (**Figures 1A, B**). PCR analysis confirmed the integration of the *BcSK* transgene into the transgenic lines, as evidenced by the presence of the target gene (*rbcsr*) and the hygromycin resistance gene (*hpt*) (**Figure 1C**).

RT-qPCR analysis revealed a significant upregulation of *BcSK* expression in positive transgenic lines, with expression levels increasing between 18- and 47-fold compared to the control (**Figure 1D**). Three high-expressing lines (5, 6, and 14) were selected for further analysis. HPLC-MS quantification demonstrated a substantial increase in the concentrations of key indole alkaloids, including indigo, indole, tryptanthin, and indirubin, in transgenic lines compared to controls (**Figure 1E**).

**Figure 6:**
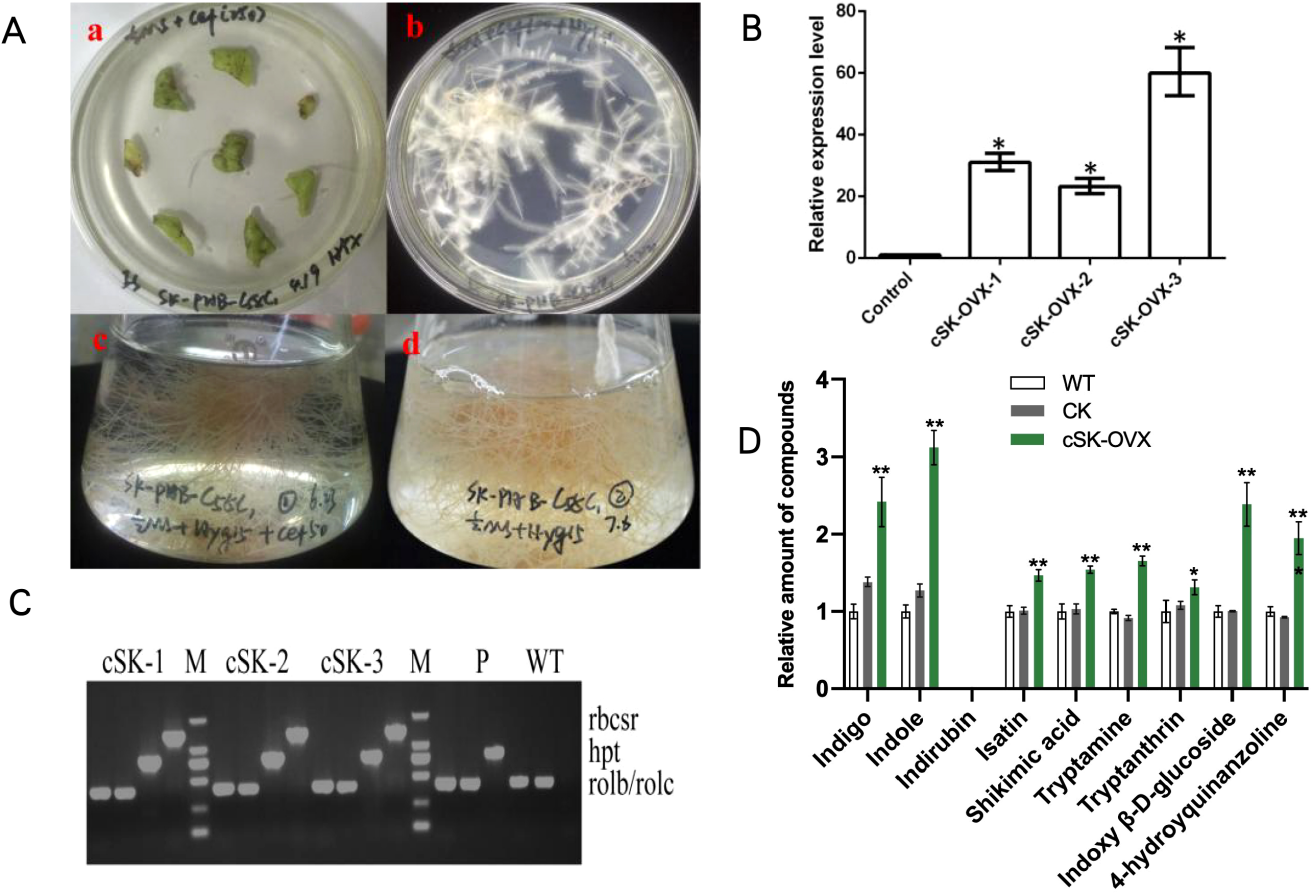
Hairy Roots of OVX-*BcSK* in *I indigotica.* **(A)** Induction and Culture of *I indigotica* Hairy Roots at Different Stages. (a) Hairy roots emerged from sterile leaves after two weeks. (b) Positive hairy roots containing the *C58C1-OVX-BcSK* vector exhibited strong growth on 1/2 MS solid medium supplemented with cefotaxime and hygromycin. (c) Liquid culture of *C58C1-OVX-BcSK* positive hairy roots in an Erlenmeyer flask. (d) Positive hairy roots continued to grow on 1/2 MS solid medium with hygromycin. **(B)** Molecular Identification of Transgenic Hairy Roots. Lane 5 and 14: DNA size marker. Lane 15–18: Engineered strain (positive control). Lane 19: Wild-type control. Lane 1–4, 6–13: *OVX-BcSK* transgenic hairy root lines. Primers *hpt, rolB*, and *rolC* were used to verify hygromycin resistance and the presence of the *Ri* plasmid. **(C)** Expression Analysis of *BcSK.* RT-qPCR analysis of *BcSK* transcript abundance in *OVX-BcSK* transgenic hairy roots. Control: *B cusia* root. **(D)** Metabolite Analysis of Transgenic Hairy Roots Metabolite quantification confirmed an increase in indole alkaloid levels. Data are presented as mean ± SEM; *P< 0.05, **P< 0.01 compared to the control.

Transgenic *I. indigotica* hairy roots were harvested after 50 days in 1/2 MS liquid culture (**Figure 6A**). Positive identification was conducted similarly to that of transgenic plants but included the detection of *rolB* and *rolC* genes, representing T-DNA fragments from the *Ri* plasmid (**Figure 6B**). This confirmed the successful transfer of the exogenous *Ri* plasmid from *A. rhizogenes* C58C1 into *I. indigotica*.

RT-qPCR analysis demonstrated a significant increase in *BcSK* expression levels in transgenic hairy roots compared to controls (**Figure 6C**). HPLC-MS analysis further confirmed elevated metabolite levels, with indole content increasing 3.12-fold compared to the control. Similarly, indigo and indoxyl β-D-glucoside levels were 2.42- and 2.38-fold higher, respectively, in *BcSK*-OVX lines compared to both wild-type (WT) and control (CK) lines (**Figure 6**).

## Discussion

Indole alkaloids constitute a diverse class of alkaloids distinguished by the presence of an indole structural moiety. Emerging evidence suggests that indole alkaloids, such as indigo and indirubin, are major constituents of *B. cusia* (Lin et al., 2019; Abouzeid et al., 2017), exhibiting higher concentrations than in other “blue” plants, including *I. indigotica, Indigofera tinctoria*, and *P. tinctorium*. Indigo and indirubin, widely recognized for their anti-inflammatory and anticancer properties in traditional Chinese medicine, are synthesized via the shikimate-derived metabolic pathway (**Supplementary Figure S1**), with indole serving as the primary precursor (Xu et al., 2020). The shikimate pathway plays a crucial role in initiating indole biosynthesis (Bentley, 1990).

Despite the significance of shikimate kinase (SK) in the shikimate pathway, no prior studies have reported its role in indole alkaloid biosynthesis in *B. cusia*. In this study, the *BcSK* gene was isolated and characterized from *B. cusia* transcriptome data. Bioinformatics analyses were employed to investigate its predicted protein structure, subcellular localization, and enzymatic properties. Additionally, its response to external stress was examined, and its functional role was validated both *in vivo* and *in vitro*.

RT-qPCR analysis demonstrated that *BcSK* is ubiquitously expressed across roots, stems, leaves, and flowers of *B. cusia*, though with varying relative expression levels. This differential expression pattern may be attributed to the shikimate pathway’s central role in the plant’s metabolic network. The pathway’s products are crucial for morphogenesis, developmental regulation, stress adaptation, and defining key plant characteristics, highlighting its importance in plant biology (Bentley, 1990).

Numerous studies have explored the regulatory mechanisms governing indole alkaloid biosynthesis genes in response to elicitor treatments. These findings contribute to uncovering the molecular mechanisms underlying indole alkaloid biosynthesis, laying the foundation for future metabolic engineering approaches (Li et al., 2023). In this study, the expression pattern of *BcSK* was analyzed following exposure to defense-related signaling molecules, including methyl jasmonate (MeJA) and abscisic acid (ABA), as well as the abiotic stressor ultraviolet (UV) radiation. *BcSK* expression exhibited significant variations compared to the control (**Figure 3**), suggesting its involvement in the plant’s response to abiotic stress.

It is well-established that exogenous MeJA treatment can significantly enhance the levels of bioactive compounds in many traditional Chinese medicinal plants (Jeyasri et al., 2023). Furthermore, the upregulation of *BcSK* in *B. cusia* leaves following UV irradiation suggests that its expression may be influenced by environmental factors such as geographical location, light exposure, and altitude. Subcellular localization analysis confirmed that *BcSK* is specifically targeted to chloroplasts (**Figure 4**), consistent with previous findings in *A. thaliana* (Zhang et al., 2008). This supports the hypothesis that *BcSK* plays a functional role in the biosynthesis of indole alkaloids.

The enzymatic function of *BcSK* was validated *in vitro*, confirming its catalytic activity. However, due to the complexity of the enzymatic reaction, direct quantification of substrate consumption and product formation remains challenging. Consequently, a coupled assay approach was employed to monitor NADH consumption during the redox reaction catalyzed by lactate dehydrogenase (Sutton et al., 2015). The observed redox activity confirmed that *BcSK* effectively catalyzed substrate conversion, supporting its functional role in the biosynthesis of shikimate-3-phosphate. Therefore, our enzymatic activity assessment conclusively demonstrates that ectopically expressed and purified *BcSK* is catalytically active *in vitro*.

The *BcSK*-OVE construct was successfully introduced into both regenerated plants and hairy roots of *I. indigotica*, an alternative source of indigo and indirubin. Due to the challenges associated with stable transformation in the perennial shrub *B. cusia*, *I. indigotica* serves as a practical model for investigating the biosynthetic pathways of indole alkaloids (Chen et al., 2018).

HPLC-MS analysis revealed that both indigo and indirubin accumulated in regenerated plants, while indirubin was notably absent from hairy roots. These overexpression results indicate that *BcSK* is essential for indole alkaloid biosynthesis in *B. cusia*, offering new insights into its functional role. Furthermore, *BcSK* is the only gene successfully overexpressed in transgenic *I. indigotica* to date, suggesting a unique role in plant secondary metabolism. The specific function of *BcSK* in immunity and defense mechanisms remains to be elucidated.

Future studies may employ single-cell multi-omics technology (Li et al., 2023; Sun et al., 2023) to determine potential interactions between *BcSK* and other genes or transcription factors. Such investigations could further clarify the mechanisms by which *BcSK* promotes indole alkaloid biosynthesis.

## Data Availability

The datasets presented in this study can be found in online repositories. The names of the repository/repositories and accession number(s) can be found in the article/**Supplementary Material**.

## Supplementary material

Supplementary Figure S1 Prediction of the biosynthetic pathway of effective components in *B. cusia*. The pink area are the substrate and product of the catalytic reaction of SK. SK, shikimate kinase. AS, anthranilate synthase. IGS, indole- 3-glycerol phosphate. TSA, tryptophan synthase alpha. CYP, cytochrome P450 monooxygenase. UGT, uridine glycosyltransferase. Dashed arrows show the multiple steps in the biosynthetic pathway.

Supplementary Figure S2 Schematic representation of the *BcSK-* GFP vector.

Supplementary Figure S3 Schematic representation of the *BcSK*- pGEX vector.

Supplementary Figure S4 Schematic representation of the *BcSK* - PHB vector.

Supplementary Figure S5 Bioinformatics analysis of BcSK. **(A)** Prediction of the secondary structure of the BcSK protein. **(B)** Prediction of the transmembrane domain of the BcSK protein. **(C)** Prediction of hydrophobic/hydrophilic regions of the BcSK protein. **(D)** Signal peptide prediction for the BcSK protein.

Supplementary Figure S6 Cloning the ORF sequence of *BcSK* gene and its tertiary structure. **(A)**. Functional domain of BcSK, **(B)**. Nucleic acid and amino acid sequences, the underlined parts are the corresponding functional domains). **(C)**. Cloning product of *BcSK*. **(D)**. Prediction of the tertiary structure of BcSK protein.
