# Supplementary figures and images for: Molecular cloning and functional characterization of the shikimate kinase gene from Baphicacanthus cusia
Source: Front Plant Sci. 2025 Apr 25;16:1560891. doi: 10.3389/fpls.2025.1560891 (PMC12062003; doi:10.3389/fpls.2025.1560891)

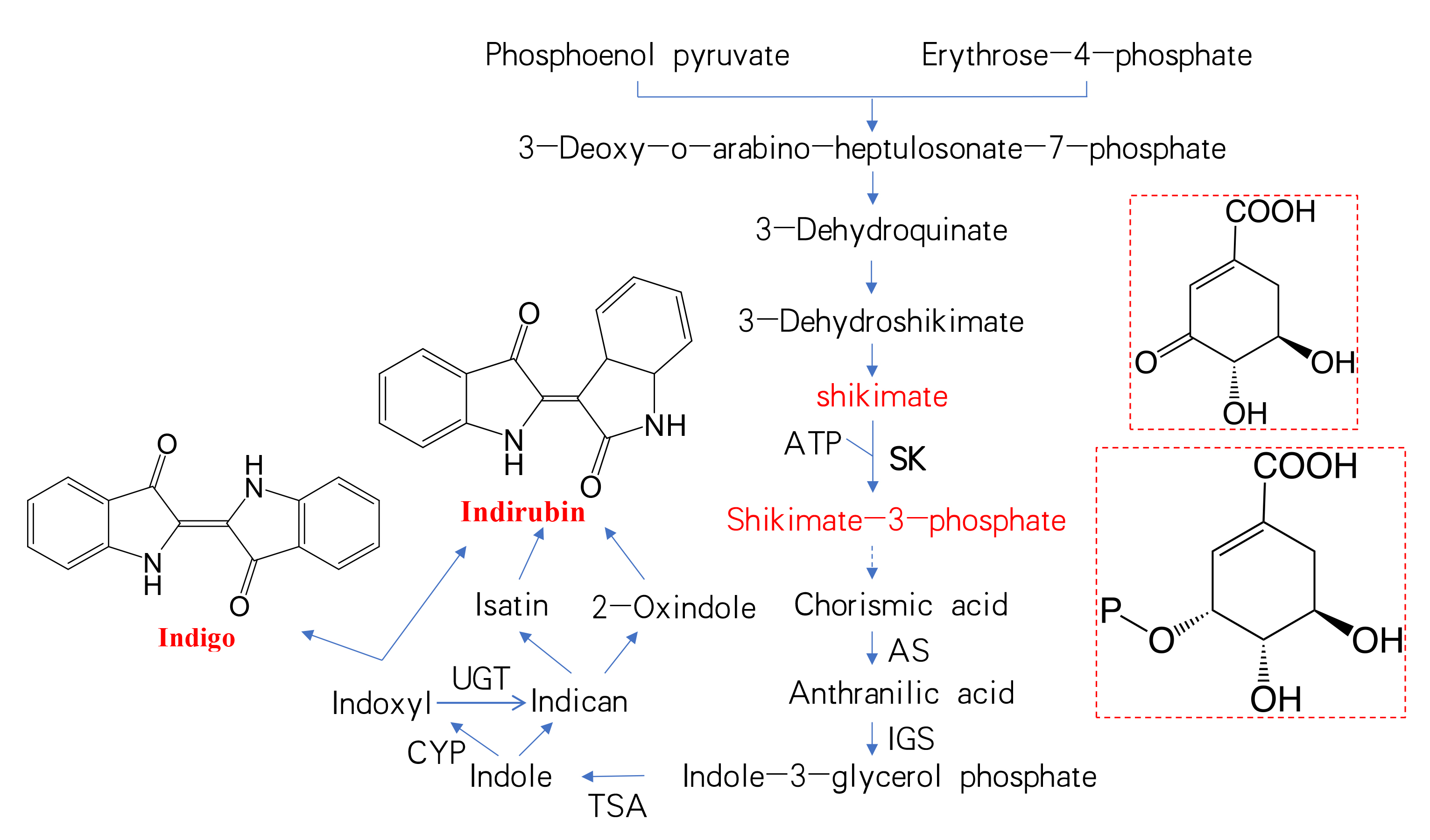

Supplement: Supplementary Figure S1 — Prediction of the biosynthetic pathway of effective components in B. cusia. The pink area are the substrate and product of the catalytic reaction of SK. SK, shikimate kinase. AS, anthranilate synthase. IGS, indole- 3-glycerol phosphate. TSA, tryptophan synthase alpha. CYP, cytochrome P450 monooxygenase. UGT, uridine glycosyltransferase. Dashed arrows show the multiple steps in the biosynthetic pathway. [file Image1.jpeg]

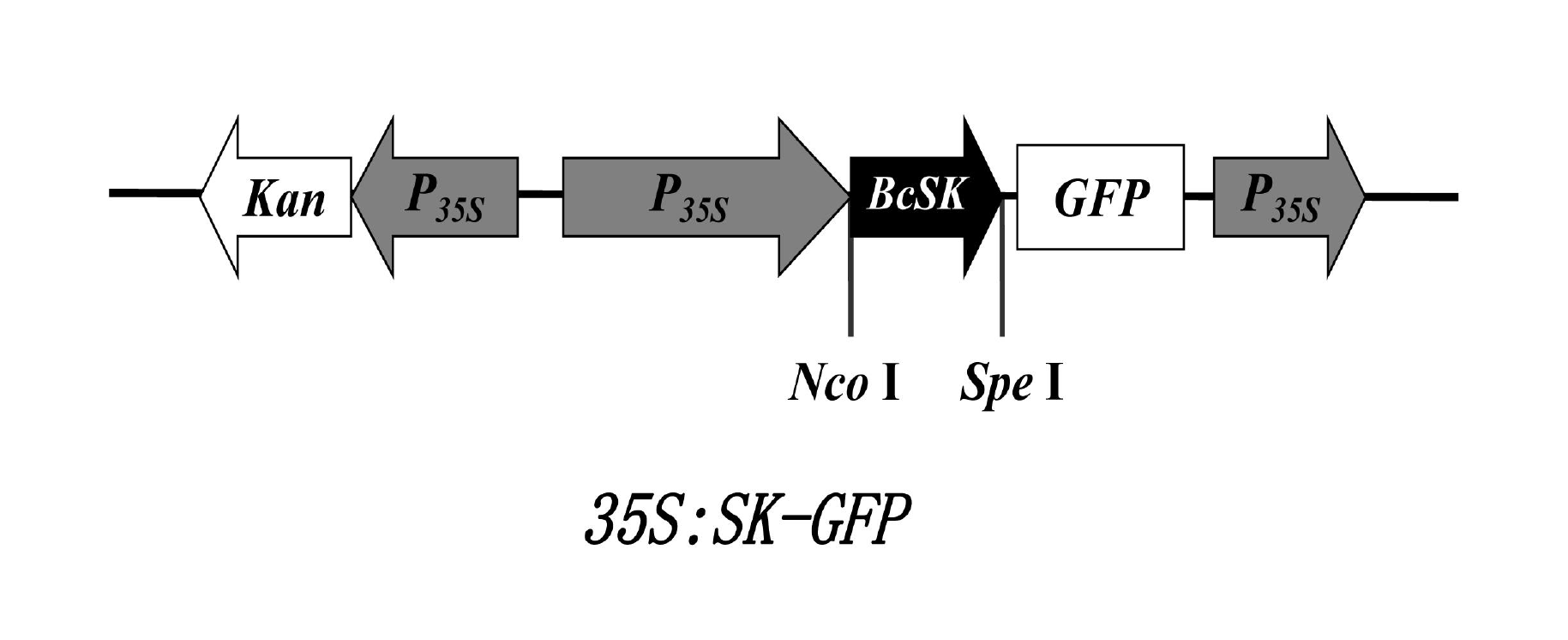

Supplement: Supplementary Figure S2 — Schematic representation of the BcSK- GFP vector. [file Image2.tif]

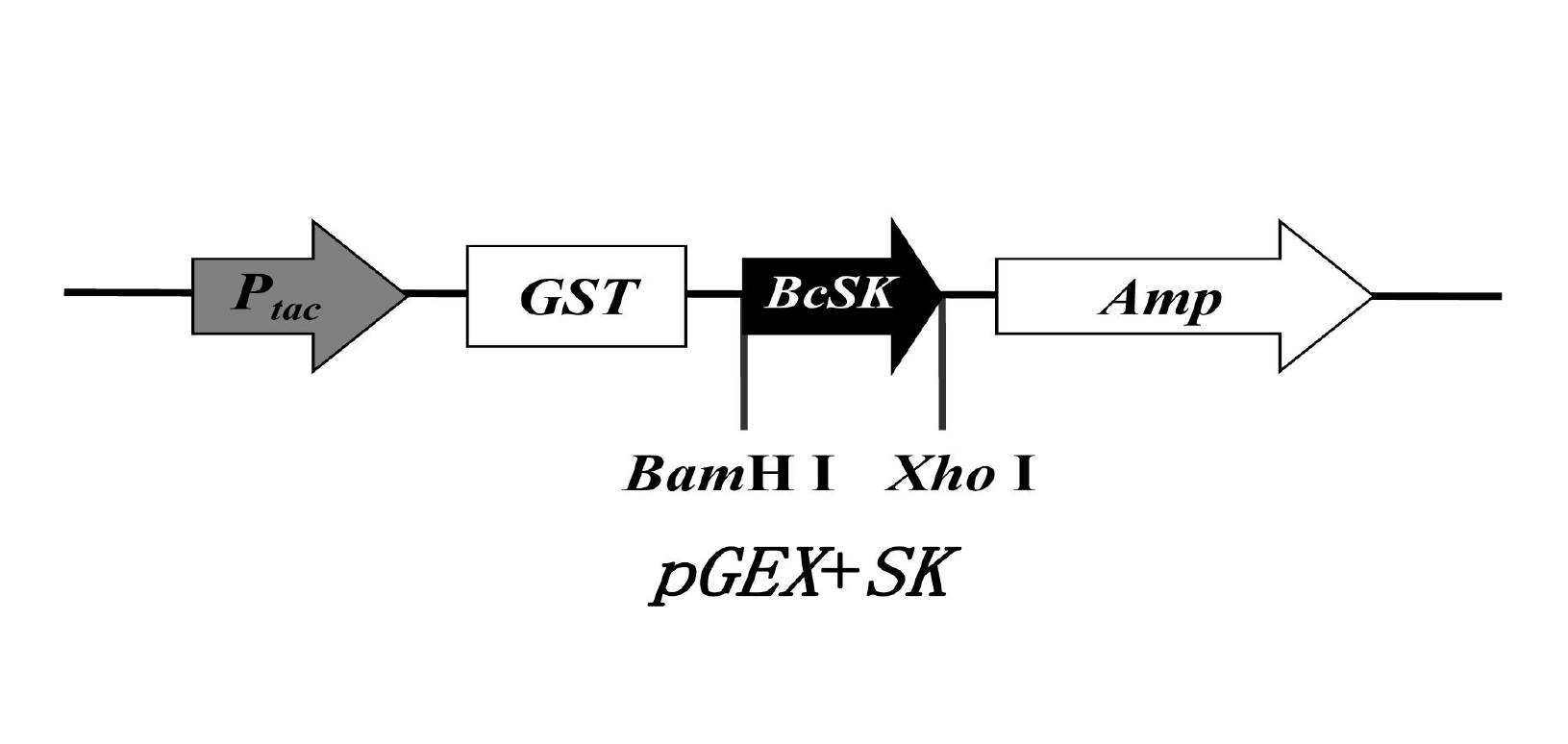

Supplement: Supplementary Figure S3 — Schematic representation of the BcSK- pGEX vector. [file Image3.tif]

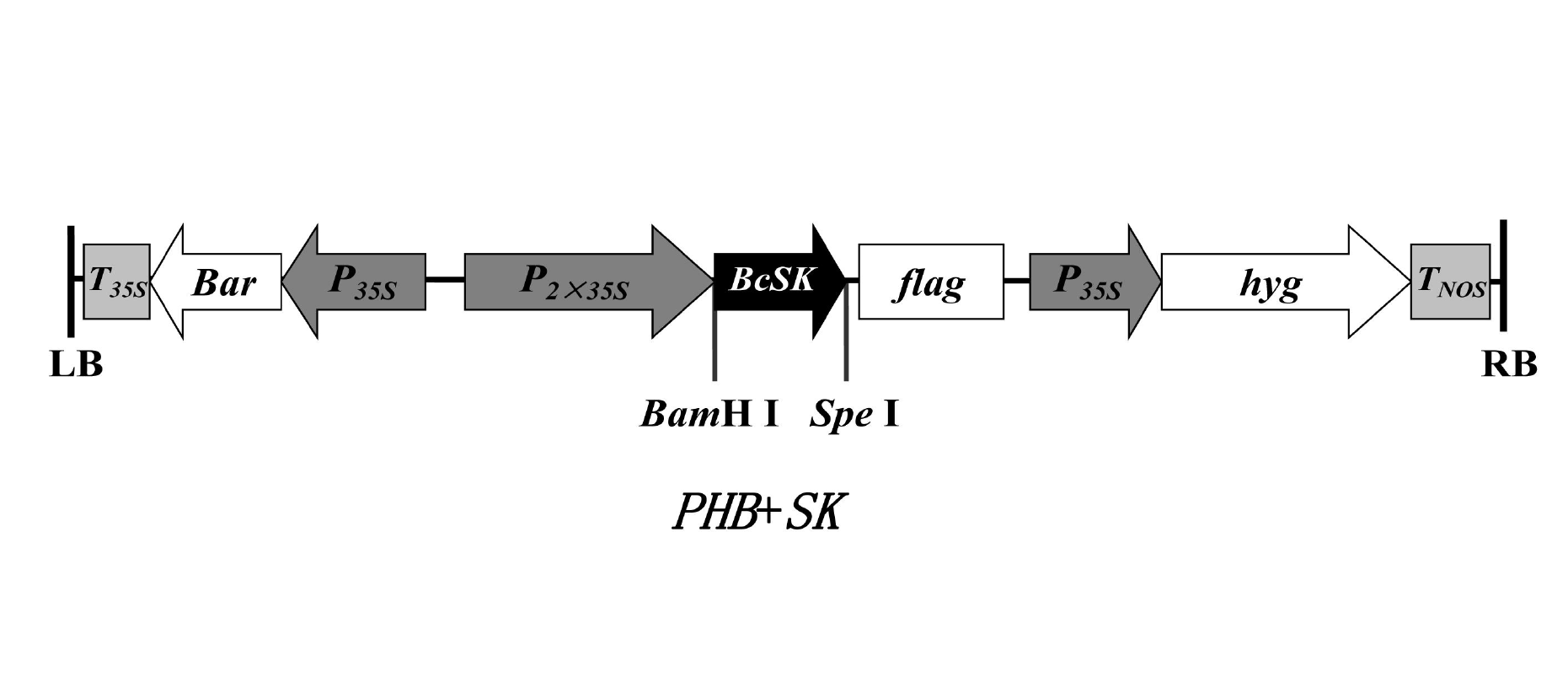

Supplement: Supplementary Figure S4 — Schematic representation of the BcSK - PHB vector. [file Image4.tif]

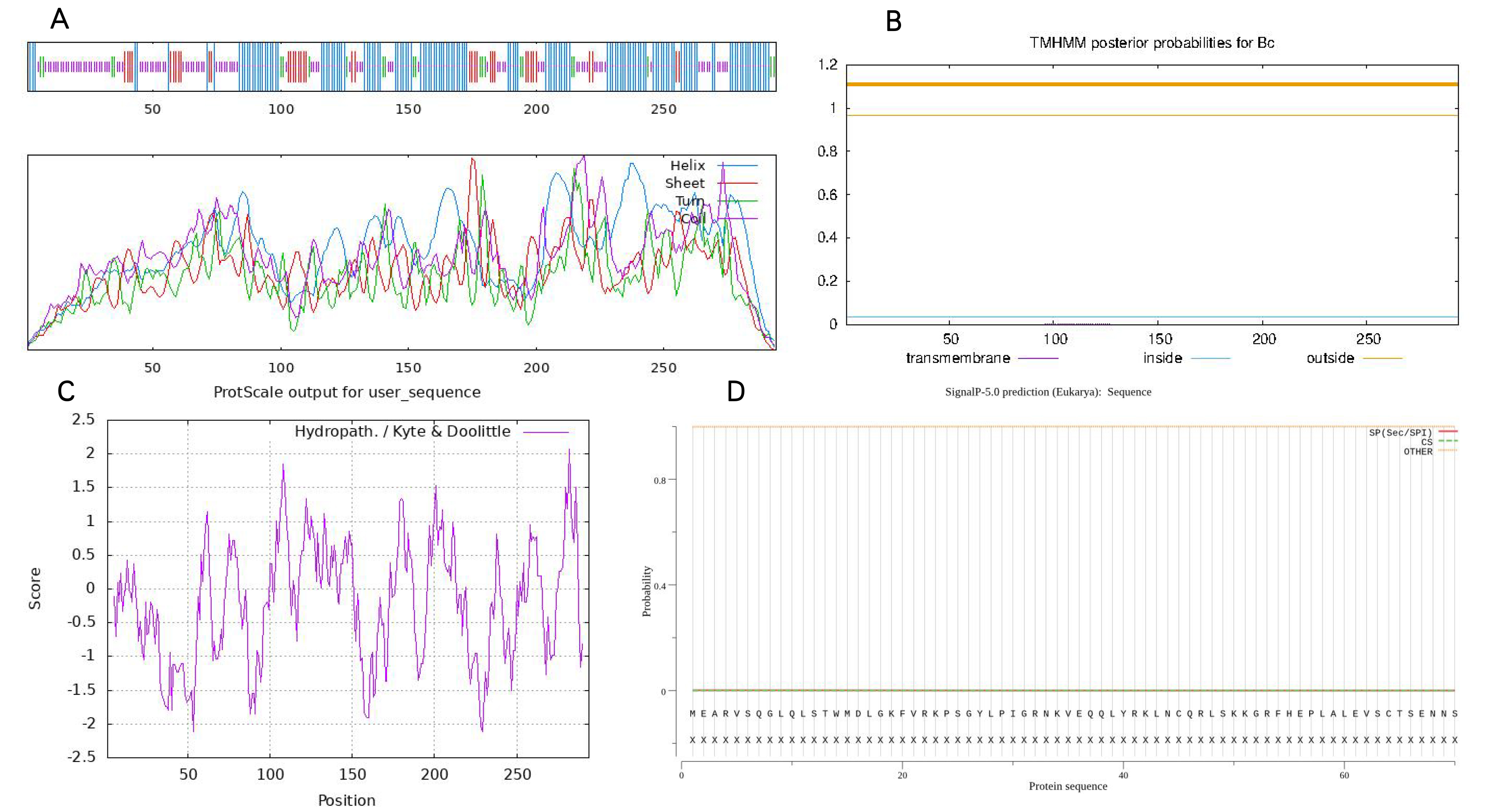

Supplement: Supplementary Figure S5 — Bioinformatics analysis of BcSK. (A) Prediction of the secondary structure of the BcSK protein. (B) Prediction of the transmembrane domain of the BcSK protein. (C) Prediction of hydrophobic/hydrophilic regions of the BcSK protein. (D) Signal peptide prediction for the BcSK protein. [file Image5.jpeg]

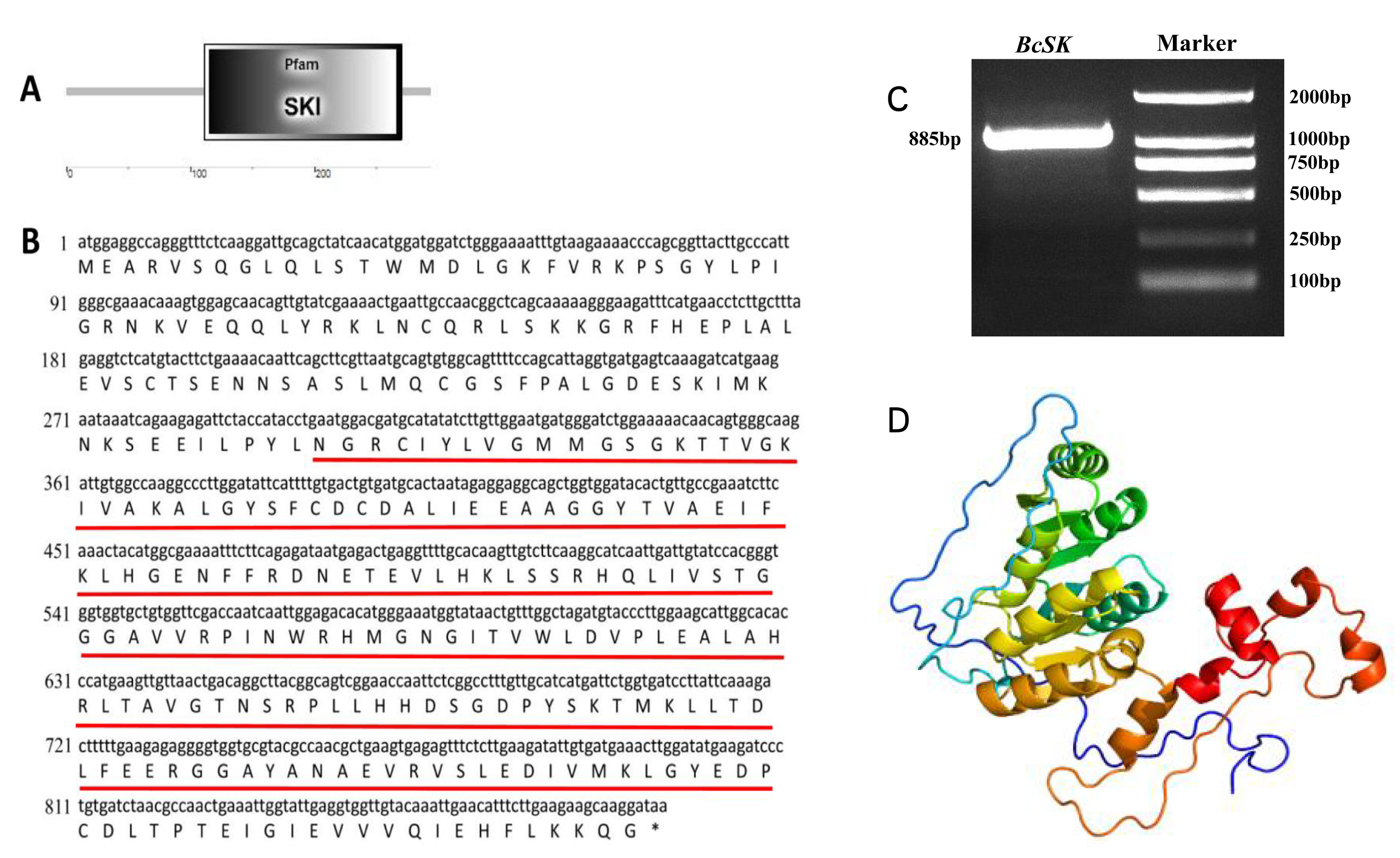

Supplement: Supplementary Figure S6 — Cloning the ORF sequence of BcSK gene and its tertiary structure. (A). Functional domain of BcSK, (B). Nucleic acid and amino acid sequences, the underlined parts are the corresponding functional domains). (C). Cloning product of BcSK. (D). Prediction of the tertiary structure of BcSK protein. [file Image6.jpeg]
