# Supplementary material for: Molecular cloning and functional characterization of the shikimate kinase gene from Baphicacanthus cusia
Source: Front Plant Sci. 2025 Apr 25;16:1560891. doi: 10.3389/fpls.2025.1560891 (PMC12062003; doi:10.3389/fpls.2025.1560891)
Supplement: Supplementary file 7 [file DataSheet1.docx]

Additional file 3: Data Set S1. The gene sequences used in the article.

>Bc c87764_g1_i1 SK

ATGGAGGCCAGGGTTTCTCAAGGATTGCAGCTATCAACATGGATGGATCTGGGAAAATTTGTAAGAAAACCCAGCGGTTACTTGCCCATTGGGCGAAACAAAGTGGAGCAACAGTTGTATCGAAAACTGAATTGCCAACGGCTCAGCAAAAAGGGAAGATTTCATGAACCTCTTGCTTTAGAGGTCTCATGTACTTCTGAAAACAATTCAGCTTCGTTAATGCAGTGTGGCAGTTTTCCAGCATTAGGTGATGAGTCAAAGATCATGAAGAATAAATCAGAAGAGATTCTACCATACCTGAATGGACGATGCATATATCTTGTTGGAATGATGGGATCTGGAAAAACAACAGTGGGCAAGATTGTGGCCAAGGCCCTTGGATATTCATTTTGTGACTGTGATGCACTAATAGAGGAGGCAGCTGGTGGATACACTGTTGCCGAAATCTTCAAACTACATGGCGAAAATTTCTTCAGAGATAATGAGACTGAGGTTTTGCACAAGTTGTCTTCAAGGCATCAATTGATTGTATCCACGGGTGGTGGTGCTGTGGTTCGACCAATCAATTGGAGACACATGGGAAATGGTATAACTGTTTGGCTAGATGTACCCTTGGAAGCATTGGCACACAGGCTTACGGCAGTCGGAACCAATTCTCGGCCTTTGTTGCATCATGATTCTGGTGATCCTTATTCAAAGACCATGAAGTTGTTAACTGACCTTTTTGAAGAGAGGGGTGGTGCGTACGCCAACGCTGAAGTGAGAGTTTCTCTTGAAGATATTGTGATGAAACTTGGATATGAAGATCCCTGTGATCTAACGCCAACTGAAATTGGTATTGAGGTGGTTGTACAAATTGAACATTTCTTGAAGAAGCAAGGATA

>Si XM_011101790.1

TTGATTCTCTTGTTTTTCACTCATAATTATGCTTAAAAATTGGAAAATATTGACCCTGGTTTAAGCGAGTTGGTGTATTGAAGTTATTGTATTTGTTTGGCATGTGTGCTCTTCTTTCCCCCCCTCTATATCTGGTCTGAGTCTATTGGAAGAGAGGGGGGGTGATATAACATGGAGGCAAAAGTTTCCCGAGGCGTGCAATTGCCCAATTGGATAAACCCAGAGAAGATTGCAAGAAAACCGAACAGTTCTTTGCAGTTTTTTCAGAAATATGGGGAACAACGGTTGTACCGAGTACCTGCTTCTTGTAATCTGCTAACTAAAAGAGGAAGATTCCATGGGCCTGCAGTTTTAAAGATCTCGTGTTCTTCTGAAAGCAGTCCAGCTTTCGTACTGGAGTCTGGAAGTTATGCAGCATCTTCTGATGAATCAAAGATCATAAAAAATAAATCAGAGGAGATTGAACCATATCTAAACGGACGGTGTATATATCTAGTTGGAATGATGGGATCTGGAAAAACAACAGTGGGCAAAGTTTTGTCGGAAACCCTGGAATATTCATTTTGTGACTGTGACACGCTGATAGAGGAGGCTGTTGGTGGAAGTACTGTGGCTGAAATATTTAAGTTATATGGCGAGAACTTCTTCAGAGATAATGAGACTGAGGTATTACACAAGTTGTCTCTGATGCACCGATTAGTTGTCTCCACGGGTGGGGGCGCTGTGGTTCGGTCAATCAATTGGAGATACATGCAAAAGGGTATCAGTGTTTGGCTAGACGTACCTTTGGAAGCCTTAGCACGGAGAATTACAGCAGTCGGAACTAATTCCCGGCCCCTATTGCATCATGAATCCGGAGATCCTTATTCAAAGACCATGAAGCGACTAACTTACCTTTTCGAAGAGAGGGGTGAAGCATATAAGAATGCTGATGTCAGAGTTTCTCTTGAAGATATTGCGGCCAAACTTGGATTGGAAGATGTATGTAATCTCACACCAACTGTAATTGCACTTGAGGTGCTTCTACAAATTGAGAATTTCTTGAAGAGATAAGGAATGAACATTATTCAATTCTTTCAATTCCATCAAATTCCATAAGTTGAGTGCTGCATTTCGATTTTCCAGCTGCGAGACGAATAATGTGAAAGATGTGGGGGTTGTCAATTAAGTGTAATAATCTACTGTAAATGTTGTATCATTGTATGACAGATGCTTTGAGTTCATCTGGATCTTGGGGCTTCTCTTTTGTAATTTTATGTAACCGAACATTGTATCTGCTATTTTTGTTTATTCAACTTGTAGTCCCATTGTTGCTCTAGCTTTGATGTTGTTCCATCTATATACCAATCAGAATTTTTTAATAC

>Si XM_011101789.1

ATAATCGTCGGTTTCCGTCATTTCCAACAACCTTAACCCCATTCAACTTTCTCATTGCATCACCAACCAAACGCACACGGGAGTTTTTTTTCAGGCTTGTATAAATACTACTCCATTTTGAAGTTGAGAGGTCTCAAAGGTCCACCTAACCCAATTCCCACTTCAACAACCACCGTCTTTGAACGTCTGAGTCTATTGGAAGAGAGGGGGGGTGATATAACATGGAGGCAAAAGTTTCCCGAGGCGTGCAATTGCCCAATTGGATAAACCCAGAGAAGATTGCAAGAAAACCGAACAGTTCTTTGCAGTTTTTTCAGAAATATGGGGAACAACGGTTGTACCGAGTACCTGCTTCTTGTAATCTGCTAACTAAAAGAGGAAGATTCCATGGGCCTGCAGTTTTAAAGATCTCGTGTTCTTCTGAAAGCAGTCCAGCTTTCGTACTGGAGTCTGGAAGTTATGCAGCATCTTCTGATGAATCAAAGATCATAAAAAATAAATCAGAGGAGATTGAACCATATCTAAACGGACGGTGTATATATCTAGTTGGAATGATGGGATCTGGAAAAACAACAGTGGGCAAAGTTTTGTCGGAAACCCTGGAATATTCATTTTGTGACTGTGACACGCTGATAGAGGAGGCTGTTGGTGGAAGTACTGTGGCTGAAATATTTAAGTTATATGGCGAGAACTTCTTCAGAGATAATGAGACTGAGGTATTACACAAGTTGTCTCTGATGCACCGATTAGTTGTCTCCACGGGTGGGGGCGCTGTGGTTCGGTCAATCAATTGGAGATACATGCAAAAGGGTATCAGTGTTTGGCTAGACGTACCTTTGGAAGCCTTAGCACGGAGAATTACAGCAGTCGGAACTAATTCCCGGCCCCTATTGCATCATGAATCCGGAGATCCTTATTCAAAGACCATGAAGCGACTAACTTACCTTTTCGAAGAGAGGGGTGAAGCATATAAGAATGCTGATGTCAGAGTTTCTCTTGAAGATATTGCGGCCAAACTTGGATTGGAAGATGTATGTAATCTCACACCAACTGTAATTGCACTTGAGGTGCTTCTACAAATTGAGAATTTCTTGAAGAGATAAGGAATGAACATTATTCAATTCTTTCAATTCCATCAAATTCCATAAGTTGAGTGCTGCATTTCGATTTTCCAGCTGCGAGACGAATAATGTGAAAGATGTGGGGGTTGTCAATTAAGTGTAATAATCTACTGTAAATGTTGTATCATTGTATGACAGATGCTTTGAGTTCATCTGGATCTTGGGGCTTCTCTTTTGTAATTTTATGTAACCGAACATTGTATCTGCTATTTTTGTTTATTCAACTTGTAGTCCCATTGTTGCTCTAGCTTTGATGTTGTTCCATCTATATACCAATCAGAATTTTTTAATAC

>Ca KX255696.1

ATGGAGGCAAAAATTGCAAGGAGTTTGCAACTCTCCCCATGGGTTAGTCCAGAAAATAGATTGCCAAAGTCGAATGGCTCGCTGAGTTTTTCCAAGTGGCATACTAAACAACAGAGAAGTCAGATTCTTATTTCCTGTCACTTTCAACCCATAAAAACCACAAGTTGGCACAGACCAGGTTCGCTGACGGCTTCATGTTCTTCTCAGAATTCTCAAGCTTCAGTTCTGCAATCTGAAAACTCTCCACCAACCCTTGATGAACTTCAAGTTTTGAAGAGTAAGTCAGAAGAGATTGAACCATATCTAAGCGGGCGATGCATATATCTTGTTGGAATGATGGGCTCTGGCAAAACAACTGTTGGCAGAATTATGGCAGAGGCACTTGGCTATTCATTTTTTGACTGTGATACACTTATAGAGCAAGCTGTTGGTGGAACAACTGTAGCTGAAATCTTTAAGCTACATGGCGAGGGCTTCTTTAGAGAAAATGAGACAGAGGTATTGCGCAAGCTTTCTTTGATGCGCGAGATTGTTGTTTCCACTGGTGGGGGTGCAGTTGTTCGGCCCATTAACTGGAAATATATGCACAAGGGCATTAGTGTGTGGTTAGATGTACCTGTGGATGCCTTAGCTAGGAGAATCTCAGCAGTAGGAACTCAATCCCGGCCCCTATTACATAATGAATCTGGAGACATGTATGCCAAGACTCTCAAACGTTTGTCTACACTTCTCGACGAGAGGGAGGATGCATACGCCAATGCAAAAGCTAGAGTTTGCCTAGAAAATATTGCTGCAAAAAGTGGATGCAATGATGTTTGCACTATAACTCCTACGAAAATTGCCATTGAGGCACTTGTACAAATCGAAAACTTCTTGAAGAAAGAAAGTGGAGAAGTGCATTGA

>In XM_019294931.1

CTTACCCACTCACCGGCTACCACCAAGTCACCAACTAAAAGTTTCCGCTTCATATAAATATAACCAATTTTGAAGCGTGAAGCACACCTACCTACCTCCACAATCTCCAGACTCCATTGCAGACAAGGAAGACCAAAGCCCGCTCATCTTTGCTTTCACCTTGTCCCTCGCCTTCCACGGTGCAGAGATAGTATAGTGACGAATTAAGGTTATTGATCAATTGAGAGGAATTGGAGGAGTAGGATTTATGGAGGCAAAAATTGCAAGGAGTTTTCAACTCACCCCATGGATTAGTTCAGAAAATAGACTGCCAAAGTCGAATGGCTCGCTGAGTTTTTCCAAGTGTCAGACCAAACATCGGAGAAGTCAGATGTTTATTTCCTGTCACTTTCAACCTATAAAAGCCAAGAGTTGGCACAGACCATGTTTGCTGACGGCTTCATGTTCTTCTCAGAATTCTCAAGCTTCTATTATGGAATCTGAAAACTCTCTACCATCCCTTGATGAAGTTCAAGTTTTGAAGAGTAAGTCAGAAGAGATTGAACCGTACCTAAGCGGGCGATGCATATATCTTGTTGGAATGATGGGCTCTGGCAAAACAACCGTTGGCAGAATTATTGCAGAGGCACTTGGCTATGCATTTTTTGACTGTGATACACTTATAGAGCAAGCTGTTGGTGGAACAAGTGTTGCTGAAATCTTTAAGCTACACGGCGAGGGCTTCTTTAGAGACAATGAGACAGAGGTCTTGCGCAAGCTTTCTTTGATGCGTGAGGTTGTTGTTTCCACTGGTGGGGGTGCGGTTGTTCGGCCCATTAACTGGAGATATATGCACAAGGGTATTAGTGTCTGGTTAGATGTACCTGTGGATGCCTTAGCTAGGAGAATTTCAGCAGTTGGAACTCATTCCCGGCCCCTATTACATAATGAATCTGGGGACATTTATGCCAAGACTCTCAAACGTTTGTCTACACTTCTGGACGAGAGGGGGGATGCATATGCCAATTCCAAAGCTAGGGTTTGCCTAGAAAATATTGCTGCAAAAAGCGGTTGCATTGATGTTTGCACTATCACTCCTATGGAGATTGCCATTGAGGCACTTGTACAAATTGGAAACTTCTTGAAGAAAGAAAGTAGAGAAGTGCATTGAGCTCCATCATATACATGCATTTTGGGTGGTAGCTCCTCTGCTTCTGTTTTTTTTTTTTTTTTTTGTTCTTCTTTTTCCTGTCCAA

>In XM_019294926.1

TCTTACCCACTCACCGGCTACCACCAAGTCACCAACTAAAAGTTTCCGCTTCATATAAATATAACCAATTTTGAAGCGTGAAGCACACCTACCTACCTCCACAATCTCCAGACTCCATTGCAGACAAGGAAGACCAAAGCCCGCTCATCTTTGCTTTCACCTTGTCCCTCGCCTTCCACGGTGCAGAGATAGTATAGTGACGAATTAAGGTTATTGATCAATTGAGAGGAATTGGAGGAGTAGGATTTATGGAGGCAAAAATTGCAAGGAGTTTTCAACTCACCCCATGGATTAGTTCAGAAAATAGACTGCCAAAGTCGAATGGCTCGCTGAGTTTTTCCAAGTGTCAGACCAAACATCGGAGAAGTCAGATGTTTATTTCCTGTCACTTTCAACCTATAAAAGCCAAGAGTTGGCACAGACCATGTTTGCTGACGGCTTCATGTTCTTCTCAGAATTCTCAAGCTTCTATTATGGAATCTGAAAACTCTCTACCATCCCTTGATGAAGTTCAAGTTTTGAAGAGTAAGTCAGAAGAGATTGAACCGTACCTAAGCGGGCGATGCATATATCTTGTTGGAATGATGGGCTCTGGCAAAACAACCGTTGGCAGAATTATTGCAGAGGCACTTGGCTATGCATTTTTTGACTGTGATACACTTATAGAGCAAGCTGTTGGTGGAACAAGTGTTGCTGAAATCTTTAAGCTACACGGCGAGGGCTTCTTTAGAGACAATGAGACAGAGGTCTTGCGCAAGCTTTCTTTGATGCGTGAGGTTGTTGTTTCCACTGGTGGGGGTGCGGTTGTTCGGCCCATTAACTGGAGATATATGCACAAGGGTATTAGTGTCTGGTTAGATGTACCTGTGGATGCCTTAGCTAGGAGAATTTCAGCAGTTGGAACTCATTCCCGGCCCCTATTACATAATGAATCTGGGGACATTTATGCCAAGACTCTCAAACGTTTGTCTACACTTCTGGACGAGAGGGGGGATGCATATGCCAATTCCAAAGCTAGGGTTTGCCTAGAAAATATTGCTGCAAAAAGCGGTTGCATTGATGTTTGCACTATCACTCCTATGGAGATTGCCATTGAGGTCCGTCTCCTTGTCTCTCTTTCTCTCTCTTTTATTTCTGTGTATATCCAATCTTTTCTTGCAATTGTCTGCATTTATAGCATTTTTTAGCATGTGAATGTGGTTTCTAGCATTTTTTACACTCTAAATCGATACATAGGCAACAATAAGTATTTGCATAGCTAACTTGGTCTCGCTCACATCCAGGCACTTGTACAAATTGGAAACTTCTTGAAGAAAGAAAGTAGAGAAGTGCATTGAGCTCCATCATATACATGCATTTTGGGTGGTAGCTCCTCTGCTTCTGTTTTTTTTTTTTTTTTTTGTTCTTCTTTTTCCTGTCCAA

>In XM_019295934.1

GGGTTGTTGAGGGAGAGGGGTTGTACACTTGTAGTCTTTGCGCAGCTGAAGCCCGACTACACCCTTTCATCAATCGATTTCGAGTCGAGGTAGTTTCAAAAGGGAGAATTCATAGGCGCTGGATCGGATCCTCTGTCGCAAATTCTTTCCTTCCACCTGTCACTTTCTCTCAAGAGATAGTGTAGTGACGAATTAAGGTTATTGATCAATTGAGAGGAGTTGGAGAAGCAGGATTTATGGAGGCAAAAATTGCAACGAGTTTGCAACTCTCCCCATGGATTAGTTCAGAAAATAGACTGCCAAAGTCCAATGGCTCGCTGAGGTTTTCCAAGTGTCAGACCAAACATCAGAGAAGTCAGATGTTAATTTCCTGTCACTTTCAACCTATAAAAGCCAAGAGTTGGCACAAACAATGTTTGCTGACGGCTTCATGTTCTTCTCAGGATTCTCAAGCTTCTATTATGGAATCTGAAAACTCTCTACCATCCCTTGATGAAGTTCAAGTTTTGAAGAGTAAGTCAGAAGAGATTGAACCGTACCTAAGCGGGCGATGCATATATCTTGTTGGAATGATGGGCTCTGGCAAAACAACCGTTGGCAGAATTATTGCAGAGGCACTTGGCTATGCATTTTTTGACTGTGATACACTTATAGAGCAAGCTGTTGGTGGAACACGTGTTGCTGAAATCTTTAAGCTACACGGCGAGGGCTTCTTTAGAGACAATGAGACAGAGGTCTTGCGCAAGCTTTCTTTGATGCGTGAGGTTGTTGTTTCCACTGGTGGGGGTGCGGTTGTTCGGCCCATTAACTGGAGATATATGCACAAGGGCGTTAGTGTCTGGTTAGATGTACCTGTGGATGCCTTAGCTAGGAGAATTTCAGTAGTTGGAACTCATTCCCGGCCCCTATTACATAATGAATCTGGGGACATTTATGCCAAGACTCTCAAACGTTTGTCTACACTTCTGGATGAAAGGGAGGATGCATATGCCAATGCCAAAGCTAGGGTTTGCCTAGAAAATATTGCTGCAAAAAGCGGTTGCATTGATGTTTGCACTATCACTCCTACGGAGATTGCCATTGAGGCACTTGTACAAATTGGAAACTTTTTGAAGAAAGAAAGTAGAGAAGTGCATTGAGCTCCATCATATACATGCATTTTGGTCAATGCTCTTCAATCTGATGACATGACTTCCAATCTGCTTCTGTTTTGTTTTTTTTTTTTTTTTTTGTTTTTTTGTTTTTTTTTTGTTCTTCTTTTTCCTGCCCAACGACAACATGTAATATACCCGGGGTGGTAGTTCAAGTGGCAAATGAGCTCTCTTTGTGGGGGAGAATTTCGGGTTCAATTCTCACAAGCGATGATTCCCCATGGGCCAGCTCCATGGCTCTCGGAGCAAACGGGGATTAATCGGGTGGACCTGGACCGATAGAGAAAGACCCGGACCACCTCGTGGTTTACACCAAAAAAAAAAAAAAAAAAA

>In XM_019295924.1

ATAAGTCTCGAGGGATTTGTGTGCAATGAATGAGGTGTTTAATCGGATTTCTGTATTTGCAGAGATAGTGTAGTGACGAATTAAGGTTATTGATCAATTGAGAGGAGTTGGAGAAGCAGGATTTATGGAGGCAAAAATTGCAACGAGTTTGCAACTCTCCCCATGGATTAGTTCAGAAAATAGACTGCCAAAGTCCAATGGCTCGCTGAGGTTTTCCAAGTGTCAGACCAAACATCAGAGAAGTCAGATGTTAATTTCCTGTCACTTTCAACCTATAAAAGCCAAGAGTTGGCACAAACAATGTTTGCTGACGGCTTCATGTTCTTCTCAGGATTCTCAAGCTTCTATTATGGAATCTGAAAACTCTCTACCATCCCTTGATGAAGTTCAAGTTTTGAAGAGTAAGTCAGAAGAGATTGAACCGTACCTAAGCGGGCGATGCATATATCTTGTTGGAATGATGGGCTCTGGCAAAACAACCGTTGGCAGAATTATTGCAGAGGCACTTGGCTATGCATTTTTTGACTGTGATACACTTATAGAGCAAGCTGTTGGTGGAACACGTGTTGCTGAAATCTTTAAGCTACACGGCGAGGGCTTCTTTAGAGACAATGAGACAGAGGTCTTGCGCAAGCTTTCTTTGATGCGTGAGGTTGTTGTTTCCACTGGTGGGGGTGCGGTTGTTCGGCCCATTAACTGGAGATATATGCACAAGGGCGTTAGTGTCTGGTTAGATGTACCTGTGGATGCCTTAGCTAGGAGAATTTCAGTAGTTGGAACTCATTCCCGGCCCCTATTACATAATGAATCTGGGGACATTTATGCCAGACTCTCAAACGTTTGTCTACACTTCTGGATGAAAGGGAGGATGCATATGCCAATGCCAAAGCTAGGGTTTGCCTAGAAAATATTGCTGCAAAAAGCGGTTGCATTGATGTTTGCACTATCACTCCTACGGAGATTGCCATTGAGGCACTTGTACAAATTGGAAACTTTTTGAAGAAAGAAAGTAGAGAAGTGCATTGAGCTCCATCATATACATGCATTTTGGTCAATGCTCTTCAATCTGATGACATGACTTCCAATCTGCTTCTGTTTTGTTTTTTTTTTTTTTTTTTGTTTTTTTGTTTTTTTTTTGTTCTTCTTTTTCCTGCCCAACGACAACATGTAATATACCCGGGGTGGTAGTTCAAGTGGCAAATGAGCTCTCTTTGTGGGGGAGAATTTCGGGTTCAATTCTCACAAGCGATGATTCCCCATGGGCCAGCTCCATGGCTCTCGGAGCAAACGGGGATTAATCGGGTGGACCTGGACCGATAGAGAAAGACCCGGACCACCTCGTGGTTTACACCAAAAAAAAAAAAAAAAAAA

>Ip JQ256515.1 TTTATGGAGGCAAAAATTGCAAGGAGTTTGCAACTCTCCCCATGGATTAGTTCAGAAAATAGACTGCCAAAGTCGAATGGCTCGCTGAGTTTTTCCAAGTGTCAGACCAAACATCGGAGAAGTCAGATGTTTATTTCCTGTCACTTTCAACCTATAAAAGCCAAGAGTTGGCACAGACCATGTTTGCTGACGGCTTCATGTTCTTCTCAGAATTCTCAAGCTTCTATTATGGAATCTGAAAACTCTCTACCATCCCTTGATGAAATTCAAGTTTTGAAGAGTAAGTCAGAAGAGATTGAACCGTGCCTAAGCGGGCGATGCATATATCTTGTTGGAATGATGGGCTCTGGCAAAACAACCGTTGGCAGAATTATTGCAGAGGCACTTGGCTATGCATTTTTTGACTGTGATACACTTATAGAGCAAGCTGTTGGTGGAACAAGTGTTGCTGAAATCTTTAAGCTACACGGCGAGGGCTTCTTTAGAGACAATGAGACAGAGGTCTTGCGCAAGCTTTCTTTGATGCGTGAGGTTGTTGTTTCCACTGGTGGGGGTGCGGTTGTTCGGCCCATTAACTGGAGATATATGCACAAGGGCATTAGTGTCTGGTTAGATGTACCTGTGGATGCCTTAGCTAGGAGAATTTCAGCAGTTGGAACTCATTCCCGACCCCTATTACATAATGAATCTGGGGACATTTATGCCAAGACTCTCAAACGTTTGTCTACACTTCTGGACGAGAGGGGGGATGCATATGCCAATTCCAAAGCTAGGGTTTGCCTAGAAAATATTGCTGCAAAAAGCGGTTGCATTGATGTTTGCACTATCACTCCTACGGAGATTGCCATTGAGGCACTTGTACAAATTGGGAACTTCTTGAAGAAAGAAAGTAGATA

>Fs DQ166524.1

TGAAGATTGATTTGAAGTTATTAGAGAAAATCAATGGACGGTAAAGTTGCAAATGGTTTGGTGGTTTCGCCGCGGATCGGTTCGGAGAGATTCGCGAGGAGAACCTGTGGTTCCGTACGGGTTTCTCGGCGATTCAGAGAGCAAGATAGGCTTCCGGTGCTAGTCTCTGCTCAGCTTCAGGATAAAACAAGGAATTCGAATTGGCACAAAACGGCGTCATTGGAGGTTTCCTGTTCTTATAAGAACTTTCCAGCTTCAGTTTTGGAATCTGGAGGTATTCATGCTCCTTTTGATGATGCTCTGATTTTGAAGAATAAGTCACAAGAGATCGAGCCATATTTAAGTGGACGCTGTATATATCTTGTTGGCATGATGGGATCTGGAAAAACAACAGTGGGCAAGGTTTTGTCACAAGTACTTAGCTATGCCTTTTTTGATAGTGACACGTTGGTGGAGCAGGATGTTGATGCAAATTCTGTTGCTGAAATATTCAATCTCTATGGAGAGGGCTTCTTCAGAGATAAGGAGACTGAGGTACTGCGAAAGCTGTCTTTGATGCATCGACTTGTTGTTTCTACTGGTGGAGGTGCAGTTGTTCGGCCCATCAACTGGAAATATATGCAGAAGGGGATAAGTGTCTGGTTAGATGTTCCTTTGGAAGCCTTGGCTCGTAGAATTGCAGCTGTAGGAACTGGTTCTCGCCCCCTTTTGCATCATGATTCGGGTGATGCATACACAAAGACTTTCATGCGCCTGACTTCTCTTATGGAGGAGAGGAGTGAAGCATATGCAAATGCAAATGCGCGGGTTTCCTTGGAAGACGTTGCAGCTAAACTAGGACACAGAGATGTGTCCAATCTCACGCCCACTGCCATAGCAATTGAGGCACTTGAACAAATCGAGGGCTTTCTAAAGGAAGAGAATGGAGATTTTGCATTGTAGTCCAAGGAGCTTATATGTATGCGTAATCACTAGTGAATTC

>Ai XM_016334477.1 ACGTAGATGGCAAAGAGCAAACCAACCAACCATGTTCGGAGTTGTCAAAAATCATCCTACTGTTGACTCCCTATGGCGATGAAAAGTCAAAAAAGCCTTCACAGGTGCCAAAAAGCGTGAAACCTAATTACTTCGTTTCTTCTCCCCCAGCCCCTTTCTCCCTTCCTTTCTTTCATTCAGAAACAAATTGAGCCAAAAAAAAAAAGCAGTGGCACCCGTCCGTCGCACTCAAAGCCGTCGTCCGTCCGTCCCTCCGTCCATCTAGCTTCCCTCGTGCTCCAAGTCTCCAACCCTGTTCGCTCTCACCGATCGCAGCTTCTTCCTCGAGGTCAGCTCGGCGTCCGACGTCTTCGTCTGCTCAGCCGCGCTTCCGGCCCAAACCATTGAATCCCTCTTTCTTTGCGGATTGAATTTCTGCCCTCTTGCTTCTCGTTCGATCTGGCAGAGTGCTCTTTTTCCACCCCCAATCCATTCTGCTAACCGCATCACAGAGCACTAGTTGCGGAACTCTGCCTCGCTGCGCCGAGGAGCACGACCTTGAACTTCCGCCGCCGCCGCCGCCTCCGTCATCTTCTGAACAATATGTTTGTTCGGTATCCGCCCTTGGAAGTTGGCTCGTGACTCGTGAGTCTTCAGTGGTGAAGTGAAAAAGAAGGAATAACAGTGGTCGTCGTCTGGTCTTCGTCTGCTCGCCGTCGCGCTCAACTGCTCTCCGTCATTGTGTGCCTAGACTGCTCTCAGATCGAAGATCGAAAATTAAGAGGGGATAAAGGACAGAACTTGTCGTTGCGATTGGCGTAAATTTTCAATACAAGTGTTGAGACGAAGTTATCATGGATGTGAAAGCTGCGAAAGCGTTGCAACTTTCAGCTCTGCTTCAACCGGAGAAGATTCGGTCAAAACCCAGTGGCGCTTCTTCTCTGAGCATATCACGTGAGCCGCACAAAAAGTTTCTCCGGGTTTCGGTTTCGGTTAAGTTGCAGCCTGTTAGAACTTCCACGATCCGGCGCAGGGTAGCACCTTTGGAGGTTACTCGTTCATACGAAAATATCCCAGCTTCGACATTGGAATCTGGGACCTGTCATGCTCTTCTTGATGAAGAGTTGATTTTGAAGAGTAGATCACAAGAGATCGATCCATATTTAAATGGACGCTGTATATATCTTGTTGGAATGATGGGATCTGGGAAGACAACTGTGGGGAAGATTATGGCACAAGTGCTTGGCTATTCTTTCTGTGATAGTGATGCATTGGTGGAGGAGGAGGTTGGAAATTCTGTAGCTGATATATTCAAGCACTATGGAGAAGCTTTCTTTCGCGATAAAGAGACCGAGGTATTGCATAAACTGTCCCTGATGCGTAGATTTGTTATTTCTACTGGTGGAGGTGCTGTTATCAGGCCCATCAATTGGAAACATATGCACAAGGGCGTCAGTGTTTGGTTGGATGTACCGCTGGAAGCCTTGGCACAGAGAATTGCAGCTGTAGGAACTAATTCTCGCCCCCTTCTACATTATGAAGCAGGAGATGCATACACCCGGGCTCTCCTGAAGTTATCTGCTCTTTTTGAAGAAAGAGGTGATTCATATGCCAATGCCAATGCCAGGGTCTCATTGGAAAAGATAGCGGCAAAACTGGGCCAAAGAGATGTGTCCAAACTGTCACCAACTGCTATTGCACTTGAGGCATTGGAACAAATTGAAGTCTTTTTGAAGGAAGAAGACAACAATTACGCAGAACGCTAATAAAAACACAGACAAGCATTGTTGACGTGTTCATATTTTTTCCGTTATATGCAATCAAGGCCATGATAATATCGGGCAATGTCAATATGTTGAGTTAAAAAAAAAAAGTGATGAAACCTGTCATATGTTTCTGCATAATTTGTGTTGGCATTTAGAATAAGCTGAGAAGCTACCAGCAGTGTTTATGTTTTGTTTATTGTTGATGTAAATATATATTTGTAGTCGAGTTTGGTTGATCTGGTTATGTATTACTTC

CTCCAAGAGGATTTTTATGTTGTAAAGTGGTCCGGTTTATTTATTTTTAAA

>Ai XM_016334476.1

ACGTAGATGGCAAAGAGCAAACCAACCAACCATGTTCGGAGTTGTCAAAAATCATCCTACTGTTGACTCCCTATGGCGATGAAAAGTCAAAAAAGCCTTCACAGGTGCCAAAAAGCGTGAAACCTAATTACTTCGTTTCTTCTCCCCCAGCCCCTTTCTCCCTTCCTTTCTTTCATTCAGAAACAAATTGAGCCAAAAAAAAAAAGCAGTGGCACCCGTCCGTCGCACTCAAAGCCGTCGTCCGTCCGTCCCTCCGTCCATCTAGCTTCCCTCGTGCTCCAAGTCTCCAACCCTGTTCGCTCTCACCGATCGCAGCTTCTTCCTCGAGGTCAGCTCGGCGTCCGACGTCTTCGTCTGCTCAGCCGCGCTTCCGGCCCAAACCATTGAATCCCTCTTTCTTTGCGGATTGAATTTCTGCCCTCTTGCTTCTCGTTCGATCTGGCAGAGTGCTCTTTTTCCACCCCCAATCCATTCTGCTAACCGCATCACAGAGCACTAGTTGCGGAACTCTGCCTCGCTGCGCCGAGGAGCACGACCTTGAACTTCCGCCGCCGCCGCCGCCTCCGTCATCTTCTGAACAATATGTTTGTTCGGTATCCGCCCTTGGAAGTTGGCTCGTGACTCGTGAGTCTTCAGTGGTGAAGTGAAAAAGAAGGAATAACAGTGGTCGTCGTCTGGTCTTCGTCTGCTCGCCGTCGCGCTCAACTGCTCTCCGTCATTGTGTGCCTAGACTGCTCTCAGATCGAAGCTGATCCCTTCCAGTCAAATTCTGCTACCACAGATCGAAAATTAAGAGGGGATAAAGGACAGAACTTGTCGTTGCGATTGGCGTAAATTTTCAATACAAGTGTTGAGACGAAGTTATCATGGATGTGAAAGCTGCGAAAGCGTTGCAACTTTCAGCTCTGCTTCAACCGGAGAAGATTCGGTCAAAACCCAGTGGCGCTTCTTCTCTGAGCATATCACGTGAGCCGCACAAAAAGTTTCTCCGGGTTTCGGTTTCGGTTAAGTTGCAGCCTGTTAGAACTTCCACGATCCGGCGCAGGGTAGCACCTTTGGAGGTTACTCGTTCATACGAAAATATCCCAGCTTCGACATTGGAATCTGGGACCTGTCATGCTCTTCTTGATGAAGAGTTGATTTTGAAGAGTAGATCACAAGAGATCGATCCATATTTAAATGGACGCTGTATATATCTTGTTGGAATGATGGGATCTGGGAAGACAACTGTGGGGAAGATTATGGCACAAGTGCTTGGCTATTCTTTCTGTGATAGTGATGCATTGGTGGAGGAGGAGGTTGGAAATTCTGTAGCTGATATATTCAAGCACTATGGAGAAGCTTTCTTTCGCGATAAAGAGACCGAGGTATTGCATAAACTGTCCCTGATGCGTAGATTTGTTATTTCTACTGGTGGAGGTGCTGTTATCAGGCCCATCAATTGGAAACATATGCACAAGGGCGTCAGTGTTTGGTTGGATGTACCGCTGGAAGCCTTGGCACAGAGAATTGCAGCTGTAGGAACTAATTCTCGCCCCCTTCTACATTATGAAGCAGGAGATGCATACACCCGGGCTCTCCTGAAGTTATCTGCTCTTTTTGAAGAAAGAGGTGATTCATATGCCAATGCCAATGCCAGGGTCTCATTGGAAAAGATAGCGGCAAAACTGGGCCAAAGAGATGTGTCCAAACTGTCACCAACTGCTATTGCACTTGAGGCATTGGAACAAATTGAAGTCTTTTTGAAGGAAGAAGACAACAATTACGCAGAACGCTAATAAAAACACAGACAAGCATTGTTGACGTGTTCATATTTTTTCCGTTATATGCAATCAAGGCCATGATAATATCGGGCAATGTCAATATGTTGAGTTAAAAAAAAAAAGTGATGAAACCTGTCATATGTTTCTGCATAATTTGTGTTGGCATTTAGAATAAGCTGAGAAGCTACCAGCAGTGTTTATGTTTTGTTTATTGTTGATGTAAATATATATTTGTAG

TCGAGTTTGGTTGATCTGGTTATGTATTACTTCCTCCAAGAGGATTTTTATGTTGTAAAGTGGTCCGGTTTATTTATTTTTAAA

>Ai XM_016334475.1

ACGTAGATGGCAAAGAGCAAACCAACCAACCATGTTCGGAGTTGTCAAAAATCATCCTACTGTTGACTCCCTATGGCGATGAAAAGTCAAAAAAGCCTTCACAGGTGCCAAAAAGCGTGAAACCTAATTACTTCGTTTCTTCTCCCCCAGCCCCTTTCTCCCTTCCTTTCTTTCATTCAGAAACAAATTGAGCCAAAAAAAAAAAGCAGTGGCACCCGTCCGTCGCACTCAAAGCCGTCGTCCGTCCGTCCCTCCGTCCATCTAGCTTCCCTCGTGCTCCAAGTCTCCAACCCTGTTCGCTCTCACCGATCGCAGCTTCTTCCTCGAGGTCAGCTCGGCGTCCGACGTCTTCGTCTGCTCAGCCGCGCTTCCGGCCCAAACCATTGAATCCCTCTTTCTTTGCGGATTGAATTTCTGCCCTCTTGCTTCTCGTTCGATCTGGCAGAGTGCTCTTTTTCCACCCCCAATCCATTCTGCTAACCGCATCACAGAGCACTAGTTGCGGAACTCTGCCTCGCTGCGCCGAGGAGCACGACCTTGAACTTCCGCCGCCGCCGCCGCCTCCGTCATCTTCTGAACAATATGTTTGTTCGGTATCCGCCCTTGGAAGTTGGCTCGTGACTCGTGAGTCTTCAGTGGTGAAGTGAAAAAGAAGGAATAACAGTGGTCGTCGTCTGGTCTTCGTCTGCTCGCCGTCGCGCTCAACTGCTCTCCGTCATTGTGTGCCTAGACTGCTCTCAGATCGAAGATTCACATAACTTTCTTCAATTTTAACTTTCTCTGGTTTAGCTGATCCCTTCCAGTCAAATTCTGCTACCACAGATCGAAAATTAAGAGGGGATAAAGGACAGAACTTGTCGTTGCGATTGGCGTAAATTTTCAATACAAGTGTTGAGACGAAGTTATCATGGATGTGAAAGCTGCGAAAGCGTTGCAACTTTCAGCTCTGCTTCAACCGGAGAAGATTCGGTCAAAACCCAGTGGCGCTTCTTCTCTGAGCATATCACGTGAGCCGCACAAAAAGTTTCTCCGGGTTTCGGTTTCGGTTAAGTTGCAGCCTGTTAGAACTTCCACGATCCGGCGCAGGGTAGCACCTTTGGAGGTTACTCGTTCATACGAAAATATCCCAGCTTCGACATTGGAATCTGGGACCTGTCATGCTCTTCTTGATGAAGAGTTGATTTTGAAGAGTAGATCACAAGAGATCGATCCATATTTAAATGGACGCTGTATATATCTTGTTGGAATGATGGGATCTGGGAAGACAACTGTGGGGAAGATTATGGCACAAGTGCTTGGCTATTCTTTCTGTGATAGTGATGCATTGGTGGAGGAGGAGGTTGGAAATTCTGTAGCTGATATATTCAAGCACTATGGAGAAGCTTTCTTTCGCGATAAAGAGACCGAGGTATTGCATAAACTGTCCCTGATGCGTAGATTTGTTATTTCTACTGGTGGAGGTGCTGTTATCAGGCCCATCAATTGGAAACATATGCACAAGGGCGTCAGTGTTTGGTTGGATGTACCGCTGGAAGCCTTGGCACAGAGAATTGCAGCTGTAGGAACTAATTCTCGCCCCCTTCTACATTATGAAGCAGGAGATGCATACACCCGGGCTCTCCTGAAGTTATCTGCTCTTTTTGAAGAAAGAGGTGATTCATATGCCAATGCCAATGCCAGGGTCTCATTGGAAAAGATAGCGGCAAAACTGGGCCAAAGAGATGTGTCCAAACTGTCACCAACTGCTATTGCACTTGAGGCATTGGAACAAATTGAAGTCTTTTTGAAGGAAGAAGACAACAATTACGCAGAACGCTAATAAAAACACAGACAAGCATTGTTGACGTGTTCATATTTTTTCCGTTATATGCAATCAAGGCCATGATAATATCGGGCAATGTCAATATGTTGAGTTAAAAAAAAAAAGTGATGAAACCTGTCATATGTTTCTGCATAATTTGTGTTGGCATTTAGAATAAGCTGAGAAGCTACCAGCAGTG

TTTATGTTTTGTTTATTGTTGATGTAAATATATATTTGTAGTCGAGTTTGGTTGATCTGGTTATGTATTACTTCCTCCAAGAGGATTTTTATGTTGTAAAGTGGTCCGGTTTATTTATTTTTAAA

>Ad XM_016100569.1

ACGCCGCAAGCAGTGGCACCCGTCCGACGCACTCAAAGCCGTCGTCCATCCGTCCATCTAGCTTCCCTCGTGCTCCAACTCTCCAACCCTGTTTGCTCTCACCGATCGCAGCTTCTTCCTCGAGGTCAGCTCGGCGTCCGACGTCTTCGTCTGCTCAGCCGCGCATCCGGCCCAAACCATTGAATCCCTCTTTCTTTGCGGATTGAATTTCTGCCCTCTTGCTTCTCGTTCGATCTGGCAGAGTGCTCTTTTTCCACCCCCAATCCATTCTGCTAACCGCATCACAGAGCACTAGTTGCGGAACTCTGCCTCGCTGCGCGGAGGAGCACGACCTTGAACTTCCGCCGCCGCCTCCGTCATCTTCTGAACAATATGTTTGTTCGGTCTCCGCCCTTGGAAGTTGGCTCGGGACTCGTGAGTCTTCAGTGGTGAAGTGAAAAAGAAGGAATAACAGTGGTCGTCGTCTGGTCTTCGTCGCCGTCGCGCTCAACTGCTCTCCGTCATTGTGTGCCTAGACTGCTCTCAGATCGAAGATCGAAAATTAAGAGGGGATAAAGGACAGAACTTGTCGTTGCGATTGGCGTAAATTTTCAATACAAGTGTTGAGACGAAGTTATCATGGATGCGAAAGCTGCGAAAGCGTTGCAACTTTCAGCTCTGCTTCAACCGGAGAAGATTCGGTCAAAACCCAGTGGCGCTTCTTCTCTGAGCATATCACGTGAGCCGCACAAAAAGTTTTTCCGGGTTTCGGTTTCGGTTAAGTTGCAGCCTGTTAGAACTTCCACGATCCGGCGCAGGGTAGCACCTTTGGAGGTTACTCGTTCATACGAAAATATCCCAGCTTCGACATTGGAATCTGGAACCTGTCATGCTCTTCTTGATGAAGAGTTGATTTTGAAGAGTAGATCACAAGAGATCGATCCATATTTAAATGGACGCTGTATATATCTTGTTGGAATGATGGGATCTGGGAAGACAACTGTGGGGAAGATTATGGCACAAGTGCTTGGCTATTCTTTCTGTGATAGTGATGCATTGGTGGAGGAGGAGGTTGGAAATTCTGTAGCTGATATATTCAAGCACTATGGAGAAGCTTTCTTTCGCGATAAAGAGACCGAGGTATTGCATAAACTGTCCCTGATGCGTAGATTTGTTATTTCTACTGGTGGAGGTGCTGTTATCAGGCCCATCAATTGGAAACATATGCACAAGGGCGTCAGTGTTTGGTTGGATGTACCGCTGGAAGCCTTGGCACAGAGAATTGCAGCTGTAGGAACTAACTCTCGCCCCCTACTACATTATGAAGCAGGAGATGCATACACCCGGGCTCTCCTGAAGTTATCTGCTCTTTTTGAAGAAGAGGTGATTCATATGCCAATGCCAATGCCAGGGTCTCATTGGAAAAGATAGCGGCAAAACTGGGCCAAAGAGATGTGTCCAAACTGTCACCAACTGCTATTGCACTTGAGGCATTGGAACAAATTGAAGTCTTTTTGAAGGAAGAAGACAACAATTACGCAGAACGCTAATAAAAACAGACACAAGCATTGTTGACGTGTTCATATTTTTTCCGTTATATGCAAGCAAGGCCATGATAATATCGTGCAATGTCAATATGTTTTTAATTTAAAAAAAAAAAAAAAAAAGTGATGAAACCTGTCATATGTTTCTGCATAATTTGTGTTAGCATTTAGAATAAGCTGAGGAGCTACCAGCAGTGTTTATGTTTTGTTTATTGTTGATGTATATATATATTTGTAGTCGAGTTTGGTTGATCTGGTTATGTATTACTTCCTCCAAGAGGATTTTTATGTTGTAAAGTGGTCTGGTTTATTTATTTTTAAA

>Ad XM_016100568.1

ACGCCGCAAGCAGTGGCACCCGTCCGACGCACTCAAAGCCGTCGTCCATCCGTCCATCTAGCTTCCCTCGTGCTCCAACTCTCCAACCCTGTTTGCTCTCACCGATCGCAGCTTCTTCCTCGAGGTCAGCTCGGCGTCCGACGTCTTCGTCTGCTCAGCCGCGCATCCGGCCCAAACCATTGAATCCCTCTTTCTTTGCGGATTGAATTTCTGCCCTCTTGCTTCTCGTTCGATCTGGCAGAGTGCTCTTTTTCCACCCCCAATCCATTCTGCTAACCGCATCACAGAGCACTAGTTGCGGAACTCTGCCTCGCTGCGCGGAGGAGCACGACCTTGAACTTCCGCCGCCGCCTCCGTCATCTTCTGAACAATATGTTTGTTCGGTCTCCGCCCTTGGAAGTTGGCTCGGGACTCGTGAGTCTTCAGTGGTGAAGTGAAAAAGAAGGAATAACAGTGGTCGTCGTCTGGTCTTCGTCGCCGTCGCGCTCAACTGCTCTCCGTCATTGTGTGCCTAGACTGCTCTCAGATCGAAGCTGATCCCTTCCAGTCAAATTCTGCTACAACAGATCGAAAATTAAGAGGGGATAAAGGACAGAACTTGTCGTTGCGATTGGCGTAAATTTTCAATACAAGTGTTGAGACGAAGTTATCATGGATGCGAAAGCTGCGAAAGCGTTGCAACTTTCAGCTCTGCTTCAACCGGAGAAGATTCGGTCAAAACCCAGTGGCGCTTCTTCTCTGAGCATATCACGTGAGCCGCACAAAAAGTTTTTCCGGGTTTCGGTTTCGGTTAAGTTGCAGCCTGTTAGAACTTCCACGATCCGGCGCAGGGTAGCACCTTTGGAGGTTACTCGTTCATACGAAAATATCCCAGCTTCGACATTGGAATCTGGAACCTGTCATGCTCTTCTTGATGAAGAGTTGATTTTGAAGAGTAGATCACAAGAGATCGATCCATATTTAAATGGACGCTGTATATATCTTGTTGGAATGATGGGATCTGGGAAGACAACTGTGGGGAAGATTATGGCACAAGTGCTTGGCTATTCTTTCTGTGATAGTGATGCATTGGTGGAGGAGGAGGTTGGAAATTCTGTAGCTGATATATTCAAGCACTATGGAGAAGCTTTCTTTCGCGATAAAGAGACCGAGGTATTGCATAAACTGTCCCTGATGCGTAGATTTGTTATTTCTACTGGTGGAGGTGCTGTTATCAGGCCCATCAATTGGAAACATATGCACAAGGGCGTCAGTGTTTGGTTGGATGTACCGCTGGAAGCCTTGGCACAGAGAATTGCAGCTGTAGGAACTAACTCTCGCCCCCTACTACATTATGAAGCAGGAGATGCATACACCCGGGCTCTCCTGAAGTTATCTGCTCTTTTTGAAGAAAGAGGTGATTCATATGCCAATGCCAATGCCAGGGTCTCATTGGAAAAGATAGCGGCAAAACTGGGCCAAAGAGATGTGTCCAAACTGTCACCAACTGCTATTGCACTTGAGGCATTGGAACAAATTGAAGTCTTTTTGAAGGAAGAAGACAACAATTACGCAGAACGCTAATAAAAACAGACACAAGCATTGTTGACGTGTTCATATTTTTTCCGTTATATGCAAGCAAGGCCATGATAATATCGTGCAATGTCAATATGTTTTTAATTTAAAAAAAAAAAAAAAAAAGTGATGAAACCTGTCATATGTTTCTGCATAATTTGTGTTAGCATTTAGAATAAGCTGAGGAGCTACCAGCAGTGTTTATGTTTTGTTTATTGTTGATGTATATATATATTTGTAGTCGAGTTTGGTTGATCTGGTTATGTATTACTTCCTCCAAGAGGATTTTTATGTTGTAAAGTGGTCTGGTTTATTTATTTTTAAA

>Ad XM_016100567.1

ACGCCGCAAGCAGTGGCACCCGTCCGACGCACTCAAAGCCGTCGTCCATCCGTCCATCTAGCTTCCCTCGTGCTCCAACTCTCCAACCCTGTTTGCTCTCACCGATCGCAGCTTCTTCCTCGAGGTCAGCTCGGCGTCCGACGTCTTCGTCTGCTCAGCCGCGCATCCGGCCCAAACCATTGAATCCCTCTTTCTTTGCGGATTGAATTTCTGCCCTCTTGCTTCTCGTTCGATCTGGCAGAGTGCTCTTTTTCCACCCCCAATCCATTCTGCTAACCGCATCACAGAGCACTAGTTGCGGAACTCTGCCTCGCTGCGCGGAGGAGCACGACCTTGAACTTCCGCCGCCGCCTCCGTCATCTTCTGAACAATATGTTTGTTCGGTCTCCGCCCTTGGAAGTTGGCTCGGGACTCGTGAGTCTTCAGTGGTGAAGTGAAAAAGAAGGAATAACAGTGGTCGTCGTCTGGTCTTCGTCGCCGTCGCGCTCAACTGCTCTCCGTCATTGTGTGCCTAGACTGCTCTCAGATCGAAGATTCACATAACTTTCTTCAATGTTAACTTTCTCTGGTTTAGCTGATCCCTTCCAGTCAAATTCTGCTACAACAGATCGAAAATTAAGAGGGGATAAAGGACAGAACTTGTCGTTGCGATTGGCGTAAATTTTCAATACAAGTGTTGAGACGAAGTTATCATGGATGCGAAAGCTGCGAAAGCGTTGCAACTTTCAGCTCTGCTTCAACCGGAGAAGATTCGGTCAAAACCCAGTGGCGCTTCTTCTCTGAGCATATCACGTGAGCCGCACAAAAAGTTTTTCCGGGTTTCGGTTTCGGTTAAGTTGCAGCCTGTTAGAACTTCCACGATCCGGCGCAGGGTAGCACCTTTGGAGGTTACTCGTTCATACGAAAATATCCCAGCTTCGACATTGGAATCTGGAACCTGTCATGCTCTTCTTGATGAAGAGTTGATTTTGAAGAGTAGATCACAAGAGATCGATCCATATTTAAATGGACGCTGTATATATCTTGTTGGAATGATGGGATCTGGGAAGACAACTGTGGGGAAGATTATGGCACAAGTGCTTGGCTATTCTTTCTGTGATAGTGATGCATTGGTGGAGGAGGAGGTTGGAAATTCTGTAGCTGATATATTCAAGCACTATGGAGAAGCTTTCTTTCGCGATAAAGAGACCGAGGTATTGCATAAACTGTCCCTGATGCGTAGATTTGTTATTTCTACTGGTGGAGGTGCTGTTATCAGGCCCATCAATTGGAAACATATGCACAAGGGCGTCAGTGTTTGGTTGGATGTACCGCTGGAAGCCTTGGCACAGAGAATTGCAGCTGTAGGAACTAACTCTCGCCCCCTACTACATTATGAAGCAGGAGATGCATACACCCGGGCTCTCCTGAAGTTATCTGCTCTTTTTGAAGAAAGAGGTGATTCATATGCCAATGCCAATGCCAGGGTCTCATTGGAAAAGATAGCGGCAAAACTGGGCCAAAGAGATGTGTCCAAACTGTCACCAACTGCTATTGCACTTGAGGCATTGGAACAAATTGAAGTCTTTTTGAAGGAAGAAGACAACAATTACGCAGAACGCTAATAAAAACAGACACAAGCATTGTTGACGTGTTCATATTTTTTCCGTTATATGCAAGCAAGGCCATGATAATATCGTGCAATGTCAATATGTTTTTAATTTAAAAAAAAAAAAAAAAAAGTGATGAAACCTGTCATATGTTTCTGCATAATTTGTGTTAGCATTTAGAATAAGCTGAGGAGCTACCAGCAGTGTTTATGTTTTGTTTATTGTTGATGTATATATATATTTGTAGTCGAGTTTGGTTGATCTGGTTATGTATTACTTCCTCCAAGAGGATTTTTATGTTGTAAAGTGGTCTGGTTTATTTATTTTTAAA

>Ai XM_016306523.1

CTATGGATGCGAAAGCTGCGCAAACGTTGCAACTTTCAGCTATGCTTCAACCAGAGACGATGCGGTCAAAACCCAGTGGCGCCTCTTCTCTGAGAATATCACGTGAACCGCACAAAAAGTTTCTCCGGGTTTCGGTTTCGGTTAAGTTGCAGCCTGTTAGAACTTCCACGATCCGGCGCAGGGTAGCACCTTTGGAGGTTACTCGTTCATACGAAAATATCGCAGCTTCGACATTGGAATCTGGAACCTGTCATGCTCTTCTTGATGAAGAGTTGATTTTGAAGAGTAGATCACAAGAGATCGATCCATATTTAAATGGACGCTGTATATATCTTGTTGGAATGATGGGATCTGGAAAGACAACTGTAGGGAAGATTCTGTCACAAGTGCTTTGTTATTCGTTTTTTGATAGCGATACATTGATTGAGGAGGAGGTTGATGGAACTTCAGTAGCTGATATATTCAAGCACTATGGAGAGACTTTTTTCCGTGATAAAGAGACTGAGGTGTTGCGGAAGTTGTCAATGACGCATAGACATGTTATATCCACGGGCGGAGGTGCTGTTGTGAGGCCTATTAATTGGAAATATATGCACAAAGGGGTTAGCATATGGTTGGATGTACCTGTAGAAGCATTGGCTCAGAGAATAACAGCTGTAGGAACTAATTCTCGCCCACTTCTACATTCTGAAGTAGGAGATGCATACACTAAGACTTTCATGCGTTTGTCTTCTCTTTTTGAAGAACGAAGCCAAGCATATGCAAATGCCAATGCCAAGGTCTCCTTAGAAAATATAGCAGCAAAACTGGGCCAAAAAGATGTATCAGATTTGACTCCAACTGCTATTGCAATTGAGGCTCTGGAACAAATCCTAGGCTTTCTAAAGAGTGAAGATGGATATTGCTAGCTCAAGCTGGTTATAATTTCTCTTCAAACCTCATTTAGTGCAGGAGAATATGCTTTTCCTTTGTGTTTGGAAAGAATTGATGTAATGCGGCACCAACATGGCACATATTTAATGTTCATGCACGATTAGTGTTACATAATTTGAGAGGAACTGCAAGCAGCAAGTATTCAGTATATACAGTTTTGATTTTTTCGTTCCATTATAAACACTCGTAGAGCATGTGCATAAACACTCGTAGA

>Va XM_017565665.1

CCAAAATTTGTTTGATCCACTTTGAATCATGGAGACATGAATTTTAAAGTTTGAGTGTGCCATAGTTGCCAATACAAAAAGTGGCTCTTCATTATTCGAGTCGGCCGACACCATTTTTTTACTGTACAATCACTGTCTAGTTTACTCCTGAATTCCACAGTTTCACGCTGAAGCGCCATGGAAGCTAAAGCGGTTCAAGCCTTTCAGTTTTCAACAATGACGCGTTCCACGAAGCCTGAGAGAACAGGGCCAAATGACTCTCTCAGAATGTTTGGTGGATTTAAGAAACAGCTTTTTGTTTCGTCAAAATCTTTGGTGGTTGCATGTTCGCATAACAATATTCCAGCGAGGACATTGGAATCTGAAACATTTCATGCTCCTGTTGATGAAAATTTTATTTTGAAGAGTAAATCACAAGAGATCGAGCCATATTTAAATGGGCGCTGTATATATCTTGTTGGAATGATGGGATCTGGGAAGACAACTGTGGGGAAGATATTGTCACAAGCGCTTTCTTATGCGTTTTTTGATAGTGATGCATTGGTGGAGGAGGAGGTTGATGGAACATCTGTAGCTGATATATTCAAGCACTATGGAGAGACATTTTTTCGTACTAAGGAGACTGAGATATTGCGGAAGCTGTCAATGATGCATAGAAATGTTATTTCTACCGGTGGAGGTGCTGTTGTGAGGCCCATCAATTGGAAATATATGCACCAAGGAATTAGTGTTTGGTTGGATGTACCTGTAGAGGCATTGGCTCAGAGAATAACAGCCGTAGGAACTGATTCTCGCCCACTTCTAAACTATGAATCGGGAGATGCATACACAAAGACTTTCATGCGTTTGTCTGCCCTTTTTGAAGAAAGAAGTGAAGCGTATGCAAATGCCAACGCTCGGGTCTCCTTGGAAAATATGGTAGCAAAACTGGGTCAAAGAGATGTGTCAGATTTATCTCCAACTGCTATTGCAATGGAGGCGCTAGAACAAATCAAAGACTTTCTTATGAGTGAAGATGGTTGTTAAGCACGTTCTGGCAGAATCTTCAAAACAAGCATGTTTTGGTAATGCATAATTCTCTCCCCAAATTAAAATGGATATGAGAATGGCAGTGTGGTGTATCCTTTTGTTTGGGAAAATAATTATGCATGCAGATATTCAGTGTTTATGCGTAATTTGTGTAAGCGTAATTTGACGGCATGCAGTGTTTGTAGTAATGTGAATTGGAAAAATCAGTGTGCATGCTTTATTCGCTGAGTTTGCAAGACAAGGGGTGCTGGCTCAAATCCAGCGTAATAAACAAAACTTTAATGATTGACTTCATGTTTGGTCAAACGTTTGAACTCTATTCACATATTGGACGTGAAAAAGTCGTGTTCATAATCTACATTTGAT

>Ai XM_016306522.1

TATAAGTTACCAAAAAAAATAACACTGATATATCGACTGAAACCATTTCAGTAGAAGTGAAGCCATGGAATCTAAAGCTGTTCAAAGGTTACAGTTTCCAGCCATGGTGTATTCAGATAAGGGTGGAAGAACAGTACCAAGTGGTTCTCTTAACGTGTCTTGTGGTTTTATGGAACATAAGAAGCTTCGGGTTTTTGTTTCAGTGCCTTCGACGAAGATTCAACAGAGAAAGCTCCCTTTGAAGATTGCATGTTCTAATAACAAAGTTCCAGCTTCAACATTGAAATCTGAAAGCTTTAAGACTCCTCTCAGTGAAGAGTTGATTCTGAAGAGTAAATCCAAAGAGGTGGAACCGTATTTAAGTGGACGCTGTATATATCTTGTTGGAATGATGGGATCTGGAAAGACAACTGTAGGGAAGATTCTGTCACAAGTGCTTTGTTATTCGTTTTTTGATAGCGATACATTGATTGAGGAGGAGGTTGATGGAACTTCAGTAGCTGATATATTCAAGCACTATGGAGAGACTTTTTTCCGTGATAAAGAGACTGAGGTGTTGCGGAAGTTGTCAATGACGCATAGACATGTTATATCCACGGGCGGAGGTGCTGTTGTGAGGCCTATTAATTGGAAATATATGCACAAAGGGGTTAGCATATGGTTGGATGTACCTGTAGAAGCATTGGCTCAGAGAATAACAGCTGTAGGAACTAATTCTCGCCCACTTCTACATTCTGAAGTAGGAGATGCATACACTAAGACTTTCATGCGTTTGTCTTCTCTTTTTGAAGAACGAAGCCAAGCATATGCAAATGCCAATGCCAAGGTCTCCTTAGAAAATATAGCAGCAAAACTGGGCCAAAAAGATGTATCAGATTTGACTCCAACTGCTATTGCAATTGAGGCTCTGGAACAAATCCTAGGCTTTCTAAAGAGTGAAGATGGATATTGCTAGCTCAAGCTGGTTATAATTTCTCTTCAAACCTCATTTAGTGCAGGAGAATATGCTTTTCCTTTGTGTTTGGAAAGAATTGATGTAATGCGGCACCAACATGGCACATATTTAATGTTCATGCACGATTAGTGTTACATAATTTGAGAGGAACTGGAAGCAGCAAGTATTCAGTATATACAGTTTTGATTTTTTGTTC

>Vr XM_014634477.1

TCTAGCGGCCGACACCATTTTTTTTATTGTACAATCACCACTGTCTAGTTTACTCCTGAATTCTACAGTTTCACGCTGAAGCGCCATGGAAGCTAAAGCGGTTCAAGCCTTTCAATTTTCAGCAATGATGCATTCCACGAAGTCTGAGATAACAGGGCCAAATGACTCTCTCAGAATGTTTGGTGGATTTAAGAAACAGCTTTTTGTTTCGTCAACGTTTCACTCACCAAGACCTTCAACGAGGTTACAGCGAAGGACAAGATCTTTGGTGGTTGCATGTTTGCATAACAATATTCCAGCGAGGACATTGGAATCTGAAACATTTCATGCTCCTGTTGATGAAAAGTTGATTTTGAAGAGTAAATCACAAGAGATCGAGCCATATTTAAATGGGCGCTGTATATATCTTGTTGGAATGATGGGATCTGGGAAGACAACTGTGGGGAAGATATTGTCACAAGCGCTTTCTTACGCGTTTTTTGACAGTGATGCATTGGTGGAGGAGGAGGTTGATGGAACATCTGTAGCTGATATATTCAAGCACTATGGAGAGACATTTTTTCGTACTAAGGAGACTGAGATATTGCGGAAGCTGTCAATGATGCATAGAAATGTTATTTCTACCGGTGGAGGTGCTGTTGTGAGGCCCATCAATTGGAAATATATGCACCAAGGAATTAGTGTTTGGTTGGATGTACCTGTAGAGGCATTGGCTCAGAGAATAACAGCCGTAGGAACTGATTCTCGTCCACTTCTAAACAATGAACCGGGAGATGCATACACAAAGACTTTCATGCGTTTGTCTGCCCTCTTTGAAGAAAGAAGTGAAGCGTATGCAAATGCCGACGCTCGGGTCTCCTTGGAAAATATGGTAGCAAAACTGGGTCAAAGAGATATGTCAGATTTATCTCCAACTGCTATTGCAATGGAGGCGCTAGAACAAATCAAAGGCTTTCTTATGAGTAAAGATGGCTGTTAAGCACGTTCTGGCAGAATCTTCAAAACAAGCATGTTTTGGTAATGCACAATTCTCTCTCCAAATTAAAATGGATATGAGAATGGCAGTGTATTGTATATATCCTTTTGTTTGGGAAAATAATGATGCATGCAGATATTCAGTGTT

>Ai XM_016326878.1

AACCGTGAAAGCCTTTGTCCACGTAACAAGTACGAGAAGTGAAGCCATGGAATCTAAAGCTGTTCAAAGGTTACAGTTTCCAGCCATGGTGTATTCAGATAAGGGTGGAAGAACAGTACCAAGTGGTTCTCTTAACGTGTCTTGTGGTTTTATGGAACATAAGAAGCTTCGGGTTTTTGTTTCAGTGCCTTCGACGAAGATTCAACAGAGAAAGCTCCCTTTGAAGATTGCATGTTCTTATAACAAAGTTCCAGCTTCAACATTGAAATCTGAAAGCTTTAAGACTCCTCTCAGTGAAGAGTTGATTCTGAAGAGTAAATCCAAAGAGGTGGAGCCGTATTTAAGTGGACGCTGTATATATCTTGTTGGAATGATGGGATCTGGAAAGACAACTGTAGGGAAGATTCTGTCACAAGTGCTTTGTTATTCGTTTTTTGATAGCGATACATTGATTGAGGAGGAGGTTGATGGAACTTCAGTAGCTGATATATTCAAGCACTATGGAGAGACTTTTTTCCGTGATAAAGAGACTGAGGTGTTGCGGAAGTTGTCAATGACGCATAGACATGTTATATCCACGGGCGGAGGTGCTGTTGTGAGGCCTATTAATTGGAAATATATGCACAAAGGGGTTAGCATATGGTTGGATGTACCTGTAGAAGCATTGGCTCAGAGAATAACAGCTGTAGGAACTAATTCTCGCCCACTTCTACATTCTGAAGTAGGAGATGCATACACTAAGACTTTCATGCGTTTGTCTTCTCTTTTTGAAGAACGAAGCCAAGCATATGCAAATGCCAATGCCAAGGTCTCCTTGGAAAATATAGCAGCAAAACTGGGCCAAAAAGATGTATCAGATTTGACTCCAACTGCTATTGCAATTGAGGCTCTGGAACAAATCCTAGGCTTTCTAAAGAGTGAAGATGGATATTGCTAGCTCAAGCTGGTTATAATTTCTCTTCAAACCTCATTTAGTGCAGGAGAATATGCTTTTCCTTTGTGTTTGGAAAGAATTGATGTAGTGCGGCACCAACATGGCACATATTTAATGTTCATGCACGATTAGTGTTACATAATTTGAGAGGAACTGCAAGCAGCAAGTATTCAGTATATACAGTTTTGATTTTTTCGTTCCATTATAAACACTCGTAGAGCATGTGCATAAACACTCGTAGAGCATGTGCATTGTAATATGATATGGTTATATCAGTTTTGTGTACGCTTTCTTCAGATACGTCTGCTCTTGTTGTAAAGTGACCCTAATTTCATTCTCTTCTACATGGCATA

>Ai XM_016326877.1

ACCGTGAAAGCCTTTGTCCACGTAACAAGTACGAGTAGAAGTGAAGCCATGGAATCTAAAGCTGTTCAAAGGTTACAGTTTCCAGCCATGGTGTATTCAGATAAGGGTGGAAGAACAGTACCAAGTGGTTCTCTTAACGTGTCTTGTGGTTTTATGGAACATAAGAAGCTTCGGGTTTTTGTTTCAGTGCCTTCGACGAAGATTCAACAGAGAAAGCTCCCTTTGAAGATTGCATGTTCTTATAACAAAGTTCCAGCTTCAACATTGAAATCTGAAAGCTTTAAGACTCCTCTCAGTGAAGAGTTGATTCTGAAGAGTAAATCCAAAGAGGTGGAGCCGTATTTAAGTGGACGCTGTATATATCTTGTTGGAATGATGGGATCTGGAAAGACAACTGTAGGGAAGATTCTGTCACAAGTGCTTTGTTATTCGTTTTTTGATAGCGATACATTGATTGAGGAGGAGGTTGATGGAACTTCAGTAGCTGATATATTCAAGCACTATGGAGAGACTTTTTTCCGTGATAAAGAGACTGAGGTGTTGCGGAAGTTGTCAATGACGCATAGACATGTTATATCCACGGGCGGAGGTGCTGTTGTGAGGCCTATTAATTGGAAATATATGCACAAAGGGGTTAGCATATGGTTGGATGTACCTGTAGAAGCATTGGCTCAGAGAATAACAGCTGTAGGAACTAATTCTCGCCCACTTCTACATTCTGAAGTAGGAGATGCATACACTAAGACTTTCATGCGTTTGTCTTCTCTTTTTGAAGAACGAAGCCAAGCATATGCAAATGCCAATGCCAAGGTCTCCTTGGAAAATATAGCAGCAAAACTGGGCCAAAAAGATGTATCAGATTTGACTCCAACTGCTATTGCAATTGAGGCTCTGGAACAAATCCTAGGCTTTCTAAAGAGTGAAGATGGATATTGCTAGCTCAAGCTGGTTATAATTTCTCTTCAAACCTCATTTAGTGCAGGAGAATATGCTTTTCCTTTGTGTTTGGAAAGAATTGATGTAGTGCGGCACCAACATGGCACATATTTAATGTTCATGCACGATTAGTGTTACATAATTTGAGAGGAACTGCAAGCAGCAAGTATTCAGTATATACAGTTTTGATTTTTTCGTTCCATTATAAACACTCGTAGAGCATGTGCATAAACACTCGTAGAGCATGTGCATTGTAATATGATATGGTTATATCAGTTTTGTGTACGCTTTCTTCAGATACGTCTGCTCTTGTTGTAAAGTGACCCTAATTTCATTCTCTTCTACATGGCATA

>Ai XM_016326876.1

ACCGTGAAAGCCTTTGTCCACGTAACAAGTACGAGGTCAGATAGAAGTGAAGCCATGGAATCTAAAGCTGTTCAAAGGTTACAGTTTCCAGCCATGGTGTATTCAGATAAGGGTGGAAGAACAGTACCAAGTGGTTCTCTTAACGTGTCTTGTGGTTTTATGGAACATAAGAAGCTTCGGGTTTTTGTTTCAGTGCCTTCGACGAAGATTCAACAGAGAAAGCTCCCTTTGAAGATTGCATGTTCTTATAACAAAGTTCCAGCTTCAACATTGAAATCTGAAAGCTTTAAGACTCCTCTCAGTGAAGAGTTGATTCTGAAGAGTAAATCCAAAGAGGTGGAGCCGTATTTAAGTGGACGCTGTATATATCTTGTTGGAATGATGGGATCTGGAAAGACAACTGTAGGGAAGATTCTGTCACAAGTGCTTTGTTATTCGTTTTTTGATAGCGATACATTGATTGAGGAGGAGGTTGATGGAACTTCAGTAGCTGATATATTCAAGCACTATGGAGAGACTTTTTTCCGTGATAAAGAGACTGAGGTGTTGCGGAAGTTGTCAATGACGCATAGACATGTTATATCCACGGGCGGAGGTGCTGTTGTGAGGCCTATTAATTGGAAATATATGCACAAAGGGGTTAGCATATGGTTGGATGTACCTGTAGAAGCATTGGCTCAGAGAATAACAGCTGTAGGAACTAATTCTCGCCCACTTCTACATTCTGAAGTAGGAGATGCATACACTAAGACTTTCATGCGTTTGTCTTCTCTTTTTGAAGAACGAAGCCAAGCATATGCAAATGCCAATGCCAAGGTCTCCTTGGAAAATATAGCAGCAAAACTGGGCCAAAAAGATGTATCAGATTTGACTCCAACTGCTATTGCAATTGAGGCTCTGGAACAAATCCTAGGCTTTCTAAAGAGTGAAGATGGATATTGCTAGCTCAAGCTGGTTATAATTTCTCTTCAAACCTCATTTAGTGCAGGAGAATATGCTTTTCCTTTGTGTTTGGAAAGAATTGATGTAGTGCGGCACCAACATGGCACATATTTAATGTTCATGCACGATTAGTGTTACATAATTTGAGAGGAACTGCAAGCAGCAAGTATTCAGTATATACAGTTTTGATTTTTTCGTTCCATTATAAACACTCGTAGAGCATGTGCATAAACACTCGTAGAGCATGTGCATTGTAATATGATATGGTTATATCAGTTTTGTGTACGCTTTCTTCAGATACGTCTGCTCTTGTTGTAAAGTGACCCTAATTTCATTCTCTTCTACATGGCATA

>Ai XM_016351351.1

TATAAGTTACCAAAAAAAATAACACTGATATATCGACTGAAACCATTTCAGTAGAAGTGAAGCCATGGAATCTAAAGCTGTTCAAAGGTTACAGTTTCCAGCCATGGTGTATTCAGATAAGGGTGGAAGAACAGTACCAAGTGGTTCTCTTAACGTGTCTTGTGGTTTTATGGAACATAAGAAGCTTCGGGTTTTTGTTTCAGTGCCTTCGACGAAGATTCAACAGAGAAAGCTCCCTTTGAAGATTGCATGTTCTTATAACAAAGTTCCAGCTTCAACATTGAAATCTGAAAGCTTTAAGACTCCTCTCAGTGAAGAGTTGATTCTGAAGAGTAAATCCAAAGAGGTGGAACCGTATTTAAGTGGACGCTGTATATATCTTGTTGGAATGATGGGATCTGGAAAGACAACTGTAGGGAAGATTCTGTCACAAGTGCTTTGTTATTCGTTTTTTGATAGGCGCAAAGCACGTGCATTGTAA
