# Supplementary material for: Molecular cloning and functional characterization of the shikimate kinase gene from Baphicacanthus cusia
Source: Front Plant Sci. 2025 Apr 25;16:1560891. doi: 10.3389/fpls.2025.1560891 (PMC12062003; doi:10.3389/fpls.2025.1560891)
Supplement: Supplementary file 8 [file Table1.docx]

Additional file 2: Table S1 PCR primers used in the text.

| **Primer name** | **Primer sequence (forward)** | **Primer sequence (reverse)** |
| --- | --- | --- |
| BcSK | gcctgcttgaagtgaagaga | AAATAGTTGCGGAGTTGCGG |
| M13 | TGTAAAACGACGGCCAGT | CAGGAAACAGCTATGAC |
| subSK | aaCCATGGGAatggaggccagggtttctca | aaACTAGTTCCTTGCTTCTTCAAGAAAT |
| 35S | GAGGACCTAACAGAACTCGCC | AAATAGTTGCGGAGTTGCGG |
| pGEX+SK | aaaGGATCCatggaggccagggtttctca | CCGGGAGCTGCATGTGTCAGAGG |
| pGEX | GGGCTGGCAAGCCACGTTTGGTG | CCGGGAGCTGCATGTGTCAGAGG |
| PHB-SK | aaaGGATCCatggaggccagggtttctca | aaaACTAGTTCCTTGCTTCTTCAAGAAAT |
| Rbcs | / | attaacttcggtcattagaggc |
| hpt | CGATTTGTGTACGCCCGACAGTC | CGATGTAGGAGGGCGTGGATATG |
| rolB | CGAGGGGATCCGATTTGCTT | GACGCCCTCCTCGCCTTCCT |
| rolC | TCGCCATGCCTCACCAACTCAC | CCTTGATCGAGCCGGGTGAGAA |
| IiActin | ATCCTCCGTCTTGACCTTGCT | TTTCCCGTTCTGCTGTTGTG |
| 18s-rRNA | TTCTCGGCCTTTGTTGCATC | TTGGCGTTAGATCACAGGGA |

Table S2 The websites used for online analysis in the text.

| **software name** | **website** |
| --- | --- |
| ORFfinder | www.ncbi.nlm.nih.gov/projects/gorf/ |
| ProtParam | http://web.expasy.org/protparam/ |
| SMART | http://smart.embl.de/ |
| SOPMA | https://npsa-prabi.ibcp.fr/cgi-bin/npsa_automat.pl?page=npsa_sopma.html |
| Predictprotein | <https://www.predictprotein.org/> |
| Plant-mPLoc | http://www.csbio.sjtu.edu.cn/bioinf/plant-multi/ |
