# Supplementary material for: Molecular cloning and functional characterization of the shikimate kinase gene from Baphicacanthus cusia
Source: Front Plant Sci. 2025 Apr 25;16:1560891. doi: 10.3389/fpls.2025.1560891 (PMC12062003; doi:10.3389/fpls.2025.1560891)
Supplement: Supplementary file 9 [file Table2.docx]

Additional file 7: Table S3 Liquid-mass spectrometry elution procedure.

| time | Ammonium acetate (5mM %) | acetonitrile/methanol (50/50+ Ammonium acetate 5mM %) |
| --- | --- | --- |
| 0 | 95 | 5 |
| 1 | 95 | 5 |
| 3 | 50 | 50 |
| 10 | 0 | 100 |
| 15 | 0 | 100 |

Additional file 7: Table S4 Liquid-mass spectrum parameters of 9 chemical compounds.

| Compound Name Precursor Ion Product Ion Ret Time (min) Fragmentor Collision Energy |
| --- |
| 4-hydroyquinanzoline 147 65 6 147 41  Indigo 263.1 190 6.8 188 45  Indirubin 263.1 190 11 149 41 |
| Tryptamine 161.1 144.1 5.6 66 9  Tryptanthrin 249.1 130 9.25 168 33  Indole 118.1 91 7.96 127 21  Indoxy β-D-glucoside 294.1 161 5.33 117 9  Shikimic acid 173 93 1.46 96 13  Isatin 146 118 6.5 96 9 |
